# The Role of Neutrophil-to-Lymphocyte Ratio in Risk Stratification and Prognostication of COVID-19: A Systematic Review and Meta-Analysis

**DOI:** 10.3390/vaccines10081233

**Published:** 2022-08-01

**Authors:** Ashwaghosha Parthasarathi, Sunag Padukudru, Sumalata Arunachal, Chetak Kadabasal Basavaraj, Mamidipudi Thirumala Krishna, Koustav Ganguly, Swapna Upadhyay, Mahesh Padukudru Anand

**Affiliations:** 1 Allergy, Asthma, and Chest Centre, Krishnamurthypuram, Mysore 570004, India; ashwa.partha@gmail.com; 2 Yenepoya Medical College, Yenepoya University, Mangalore 575018, India; 10194@yenepoya.edu.in; 3 Department of Respiratory Medicine, JSS Medical College, JSSAHER, Mysore 570015, India; sumalatha.a@jssuni.edu.in (S.A.); chethakkb@jssuni.edu.in (C.K.B.); 4 University Hospitals Birmingham NHS Foundation Trust, Institute of Immunology Immunotherapy, University of Birmingham, Birmingham B15 2GW, UK; thirumala.krishna@uhb.nhs.uk; 5 Unit of Integrative Toxicology, Institute of Environmental Medicine (IMM), Karolinska Institutet, 17177 Stockholm, Sweden; koustav.ganguly@ki.se

**Keywords:** COVID-19, neutrophil-to-lymphocyte ratio, systematic review, COVID-19 mortality, COVID-19 severity, COVID-19 outcomes, NLR

## Abstract

Several studies have proposed that the neutrophil–lymphocyte ratio (NLR) is one of the various biomarkers that can be useful in assessing COVID-19 disease-related outcomes. Our systematic review analyzes the relationship between on-admission NLR values and COVID-19 severity and mortality. Six different severity criteria were used. A search of the literature in various databases was conducted from 1 January 2020 to 1 May 2021. We calculated the pooled standardized mean difference (SMD) for the collected NLR values. A meta-regression analysis was performed, looking at the length of hospitalization and other probable confounders, such as age, gender, and comorbidities. A total of sixty-four studies were considered, which included a total of 15,683 patients. The meta-analysis showed an SMD of 3.12 (95% CI: 2.64–3.59) in NLR values between severe and non-severe patients. A difference of 3.93 (95% CI: 2.35–5.50) was found between survivors and non-survivors of the disease. Upon summary receiver operating characteristics analysis, NLR showed 80.2% (95% CI: 74.0–85.2%) sensitivity and 75.8% (95% CI: 71.3–79.9%) specificity for the prediction of severity and 78.8% (95% CI: 73.5–83.2%) sensitivity and 73.0% (95% CI: 68.4–77.1%) specificity for mortality, and was not influenced by age, gender, or co-morbid conditions. Conclusion: On admission, NLR predicts both severity and mortality in COVID-19 patients, and an NLR > 6.5 is associated with significantly greater the odds of mortality.

## 1. Introduction

Severe acute respiratory syndrome coronavirus 2 (SARS-CoV-2) is a novel coronavirus that was recognized in January 2020 [1]. Almost one month after its discovery, the disease spread worldwide. It is also called coronavirus disease 2019 (COVID-19) by the World Health Organization [2]. The virus has claimed over 6.19 million lives worldwide [3].

The symptoms of COVID-19 can vary. The majority of those who are infected present with mild symptoms such as fever, myalgia, cough, shortness of breath, etc. [4,5]. However, some cases develop severe symptoms such as pulmonary edema, acute respiratory distress syndrome (ARDS), and multi-organ failure (MODS), leading to death [6,7]. A point of grave concern is a patient’s rapid progression from relatively mild to severe disease [8]. Thus, it is prudent to identify severe cases as early as possible and provide timely interventions.

The identification and stratification of the severity and mortality of COVID-19 patients has been performed in various ways. The most commonly used method is oxygenation-based severity criteria, based on respiratory distress with a respiratory rate > 30/min, oxygen saturation ≤ 93% in the resting state, or arterial blood oxygen partial pressure (PaO_2_)/oxygen concentration (FiO_2_) ≤ 300 mmHg. However, various other criteria have been used in different studies. These include parameters such as the need for invasive mechanical ventilation (IMV), and various hematologic and radiological parameters.

Neutrophils are an important aspect of innate immunity. Pathogen-associated molecular patterns (PAMPs) in the virus are recognized by pattern-recognition receptors (PRRs), which initiate the production of pro-inflammatory mediators and neutrophil chemo-attractants. This signaling is crucial to initiate the inflammatory response and enhance neutrophil production and recruitment [9]. Lymphocytes, on the other hand, are the principal cells of an adaptive immune response in a viral infection. Lymphocyte levels are considered to be negatively correlated with the degree of systemic inflammation. An increase in systemic inflammation significantly decreases CD4+ T cells, increases CD8+ suppressor T cells and increases lymphocyte apoptosis [10]. 

The neutrophil-to-lymphocyte ratio (NLR) is a simple ratio of the counts of neutrophils and lymphocytes. It is a biomarker which can reflect the inflammatory status of a patient. Since it is a part of routine blood count analyses performed in most healthcare setups, it is economical. NLR as a biomarker has been used in various conditions such as tumors, pancreatitis, chronic obstructive pulmonary disease (COPD), and cardiovascular disease. It has also been used in the prognosis of infectious diseases, such as influenza virus infection and Middle East respiratory syndrome (MERS) [11,12].

A few systematic reviews have studied NLR’s potential as a biomarker for the stratification of COVID-19-related disease severity and mortality [13,14,15,16]. However, these reviews have a few shortcomings. All the above-mentioned reviews used only oxygenation-based severity criteria, without considering various other classification criteria. Furthermore, although previous studies have demonstrated that NLR values are also determined by race and ethnicity, this subgroup analysis is missing in the previous reviews [17]. They also fail to analyze other important endpoints, such as the relationship between NLR and the length of hospitalization. 

Thus, the primary objective of this review is to assess the relationship between on-admission NLR values and COVID-19-related disease severity and mortality outcomes. Additionally, we would also like to assess if this relationship changes based on the severity criteria used, the region in which the study was conducted and the length of hospitalization.

## 2. Materials and Methods

### 2.1. Protocol and Registration

The review followed Preferred Reporting Items For Systematic Review And Meta-Analysis (PRISMA) guidelines and the Cochrane Handbook [18]. The PRISMA checklist can be found in the Supplementary Material (Table S1). Only published, peer-reviewed original articles were eligible for inclusion. This review was registered in the PROSPERO database (CRD42021252100).

### 2.2. Inclusion and Exclusion Criteria

The inclusion of articles for systematic review was based on studies with original data. The studies had to contain information regarding the diagnostic and/or prognostic role of NLR in COVID-19 patients with participants aged 18 years or over. The patients in the study needed to have a confirmed COVID-19 diagnosis (diagnosed with positive reverse transcriptase polymerase chain reaction for SARS-CoV-2, from samples from either nasopharyngeal or oropharyngeal swab). The NLR values needed to be collected on admission. The exclusion criteria were case reports, previous SRs/MAs, literature reviews, conferences and theses. Original articles without sufficient information for extraction were also excluded. Only studies published in English were included. 

### 2.3. Data Extraction and Risk of Bias Assessment

The literature search was conducted in PubMed, EMBASE, MEDLINE, and SCOPUS databases for articles published between 1 January 2020 and 1 May 2021. The search used terms synonymous with “COVID-19”, “neutrophil-to-lymphocyte ratio”, “Severity”, and “Mortality”. A detailed search term description is listed in the Supplementary Material (Table S2).

Management of the collated studies was performed using the COVIDENCE systematic review software (Veritas Health Innovation, Melbourne, Australia). The results were later exported to Microsoft Excel. There were two rounds of screening for the selection of studies.

1. Title/abstract screening: Authors CB, SA, SP, and AP independently screened all the articles as per the pre-agreed criteria and consulted with PAM if there was disagreement.
2. Full-text screening: Articles identified in Step 1 were moved to full-text screening. Authors SP, RKP, and AP screened all articles independently. Articles eligible for final inclusion included a sensitivity and specificity analysis of NLR in predicting the severity and mortality of COVID-19. PAM was consulted for clarification and if there was disagreement between operators.

After the selection of studies, the following data were extracted:

1. The surname of first author, year and month of publication, sample size, study location, basic demographic data—i.e., mean age, gender, and comorbidities—and outcomes, i.e., disease severity and mortality.
2. NLR values were recorded, along with their standard deviation, and categorized based on mild or severe cases.
3. Sensitivity, specificity, area under the curve (AUC) data, ‘cut-off’ data for each outcome.

Data items were imported in a predefined format into Microsoft Excel. The risk of bias in the included studies was accessed for individual articles using the Newcastle-Ottawa Scale (NOS) [19].

### 2.4. Synthesis of Evidence

Meta-analysis was performed using Jamovi (v1.6, The jamovi project, SYD, AUS), Review Manager (RevMan) [Computer program]. Version 5.4, The Cochrane Collaboration, 2020 and OpenMeta analyst v10.12 [20]. For studies without normal distribution, median and interquartile range (IQR) were converted to mean and standard deviation (SD) using the method described by Hozo et al. [21]. Pooled standardized mean difference (SMD), along with a 95% confidence interval (95% CI), was calculated for the mean values of NLR between groups using Der Simonian–Laird random effect models. Studentized residuals (a division of the residual from the regression model with its standard deviation) and Cook’s distances were also calculated. If the values were reported as dichotomized variables, risk ratio (RR) was calculated. Due to the different definitions of severity among studies, a separate analysis was performed for each of the individual subgroups. The various criteria used for severity could be grouped under the following categories: (1) based on need for invasive mechanical ventilation (IMV); (2) based on respiratory rate and oxygenation (pulse oximetry or arterial blood gases), i.e., respiratory rate > 30 bpm, SpO_2_ < 93%, and PaO_2_/FiO_2_ ≤ 300 mmHg; (3) based on the need for intensive care unit (ICU) admission only; (4) based on hematological parameters only; (5) based on radiological parameters (Table S3). 

Additionally, a subgroup analysis was performed to assess regional variations in both severity and mortality outcomes according to the World Health Organization (WHO), which divides the world into six WHO regions. A subgroup analysis was also conducted to assess differences in the standardized mean differences in the two groups according study design. A meta regression analysis was also performed to observe any association between NLR and length of hospitalization.

A bivariate regression model with random effects was used to calculate sensitivity, specificity, and diagnostic odds ratios (DORs). Furthermore, we generated a summary receiver operating characteristic curve (SROC) to evaluate the collective accuracy of NLR. Meta-regression analysis was performed based on length of hospitalization and other probable confounders, such as age, gender, and comorbidities. This analysis was presented as bubble plots. Heterogeneity was assessed with Cochran’s Q test and I^2^ statistic. Furthermore, the stability of the pooled data estimates was evaluated using leave-one-out analysis. Publication bias and small study bias were tested using funnel plots, rank correlation test, and Egger’s test. A trim and fill analysis was conducted to correct asymmetry around the pooled estimates. A statistically significant difference was considered if two-tailed *p* < 0.05.

## 3. Results

**Figure 1:**
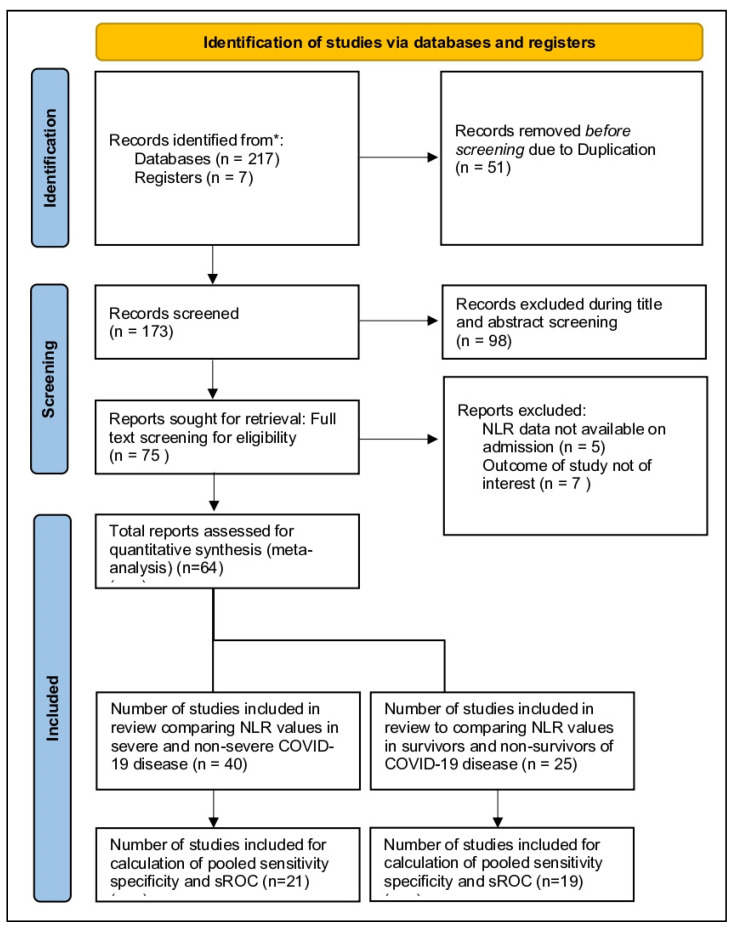
PRISMA flowchart illustrating the process by which studies were mapped out. A total of 224 records were identified, of which 64 were included in the study. * The sum of split studies does not have to equal 64, as some studies overlap in both mortality and severity aspects.

A total of 225 citations were identified from various peer-reviewed databases from 1 January 2020 to 1 May 2021. After study screening and eligibility selection, we found 64 studies consisting of 15,683, which met all our criteria. They were further divided into 40 studies that compared NLR values in severe and non-severe disease and 25 articles that compared NLR in the deceased and survivors. Twenty-five articles conducted an ROC analysis for the prognostic value of NLR to predict severe disease and nineteen articles to predict mortality outcomes (Figure 1). Most studies were retrospective and observational. The majority of included studies were conducted in China (n = 44). The median risk of bias score from NOS was found to be 7 (Table 1 and Table S6).

### 3.1. Examination of the Relationship between NLR Values and COVID-19 Severity

**Figure 2:**
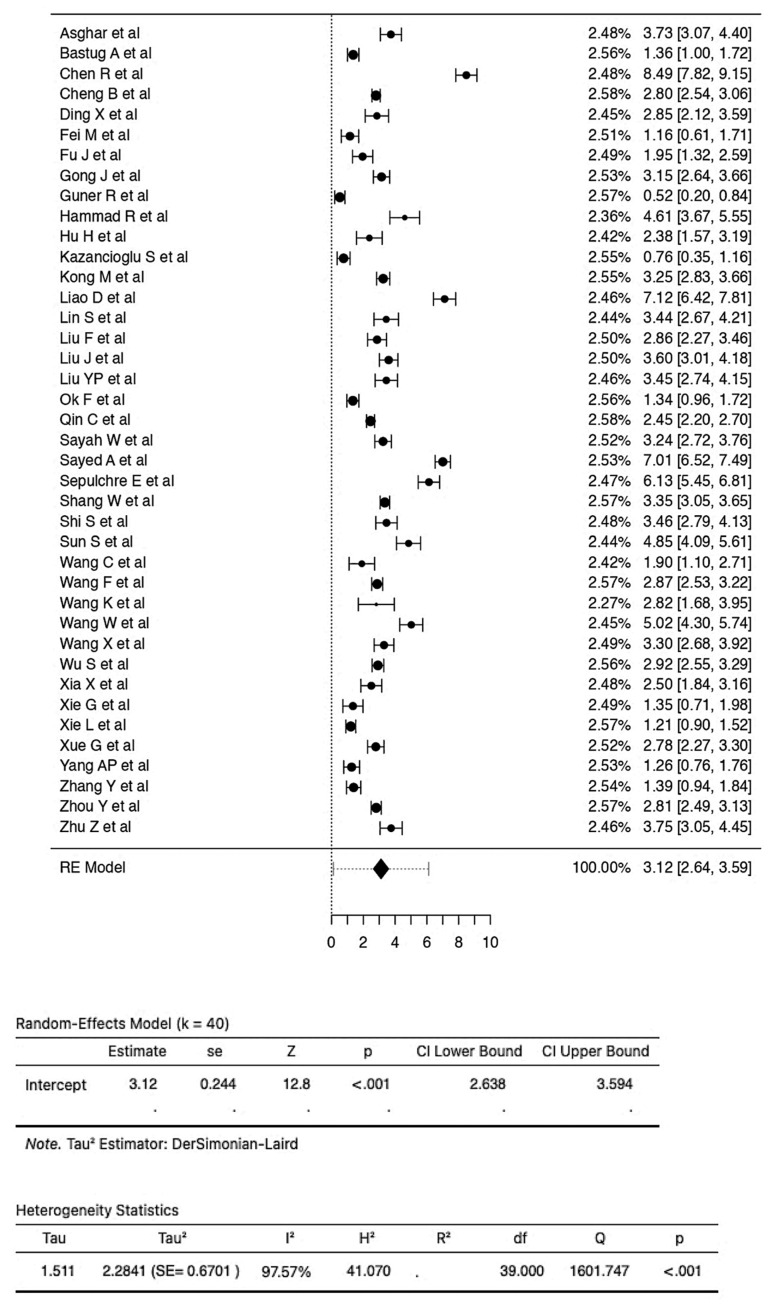
Forest plot of 40 total studies indicates the pooled SMD calculation, which was performed using the Der Simonian–Laird random effect models, observing a value of 3.12 (95% CI: from 2.64 to 3.59) between groups.

**Figure 3:**
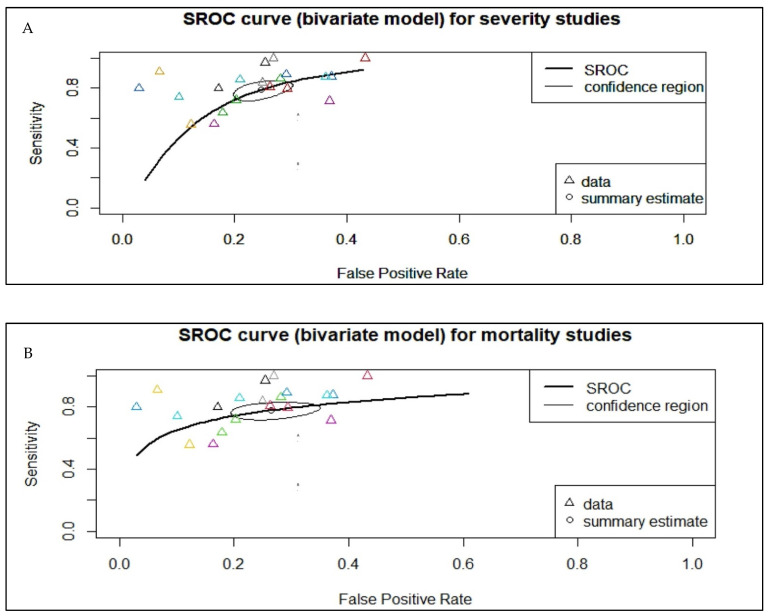
Summary receiver operating characteristic (SROC) curve, which analyzes the pooled area under the curve (AUC) for COVID-19-related outcomes. (**A**) The pooled AUC for severity studies was 0.833. (**B**) The pooled AUC for mortality studies was 0.820. The Δ stands for individual study data points while the O stands for summary estimates.

A total of 40 studies, consisting of 7332 patients, were analyzed in our review. The pooled SMD calculation was completed using the Der Simonian–Laird random effect models, which observed a value of 3.12 (95% CI: 2.64 to 3.59) between groups, with a range from 0.5224 to 8.4856. Even though the Q test showed a high degree of heterogenicity (1601.7471, *p* < 0.0001, tau^2^; = 2.2841, I^2^; = 97.5652%), the 95% prediction interval of the outcomes (ranging from 0.1156 to 6.1164) showed that SMD obtained from the individual studies were generally similar to the pooled SMD estimates. A study by Chen R et al. [29] led to a large studentized residual value (<±3.2272), making it a potential outlier. Additionally, an analysis of Cook’s distances also showed that the same study had the probability of being overly influential. However, removing this study from the analysis did not lead to a significant difference in the pooled SMD values (2.97 [2.55, 3.40]) (Figure 2). Pooled sensitivity and specificity data were calculated from the 21 studies included for the analysis. The sensitivity estimate was 80.2% (95% CI: 74.0–85.2), while the specificity estimate was 75.8% (95% CI 71.3–79.9). A SROC analysis was carried out, in which AUC was 0.833 while the DOR was 13.63 (Figure 3A and Table 2).

Different studies used varying definitions of severity. Five definitions of severity were used, and three studies did not define how they classified the disease as severe. A subgroup analysis of severity observed that NLR was significantly associated with severity for each of the definition criteria used. Even with different definitions, the effect estimates remained in the same direction as the estimated average outcome, ranging between SMD 5.64 [0.03, 11.25] and 1.99 [0.32, 3.65]. The most common severity criteria were based on oxygen status (21 studies, 3748 participants), which had an SMD of 2.76 [2.28, 3.24], followed by IMV (9 studies, 1514 participants), which had an SMD of 3.05 [2.25, 3.86], which was very close to the total estimated SMD of 3.12 [2.64, 3.59] (Figure S1A).

Subgroup analysis for COVID-19 disease severity and NLR estimates according to the different WHO regions showed that most of the studies were conducted in the Western Pacific Region (WPR), i.e., n = 31 with 5464 patients. This subgroup had an SMD of 3.07 [2.63, 3.52] and accounted for the highest weight (77.4%) among the subgroups. At the time of writing this review, a limited number of studies were available from the rest of the world, with an SMD ranging from 2.14 [0.90, 3.39] to 5.13 [2.88, 7.38] (Figure S1B).

There were minimal differences in estimated average standardized mean difference between the two subgroups when they were analyzed based on retrospective and prospective study design (SMD: 3.10 [2.59, 3.62] vs. 3.17 [2.13, 4.21]) (Figure S10).

Compared to the non-severe group, patients with severe COVID-19 were generally older and had a greater number of co-morbidities, such as diabetes mellitus, hypertension, and cardiovascular disease (Figure S6).

Pooled sensitivity and specificity data were calculated from the 21 studies included in the analysis. Sensitivity estimates were found to be 80.2% (95% CI: 74.0–85.2), while specificity estimates were 75.8% (95% CI 71.3–79.9). Summary receiver operating characteristic (SROC) analysis was carried out, in which AUC was 0.833 while the Diagnostic Odds Ratio was 13.63 (Table 2). The calculation of subgroup analysis at an NLR severity cut-off value of 4.5 showed similar AUC (0.834 and 0.833) (Table 3).

**Figure 4:**
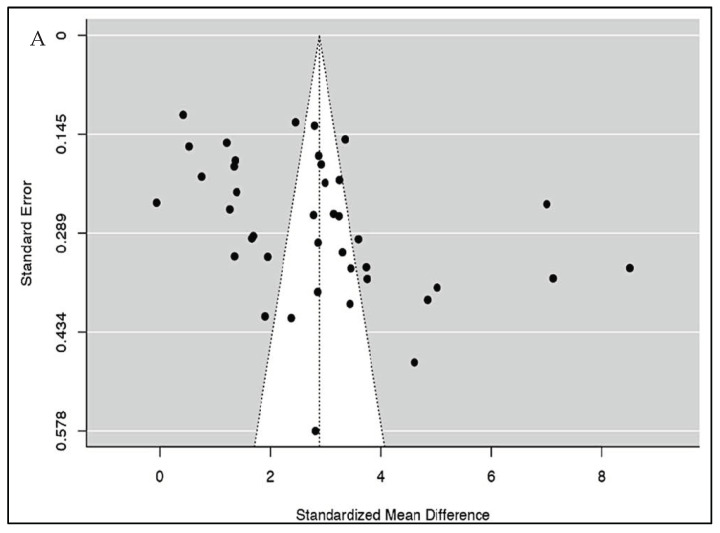

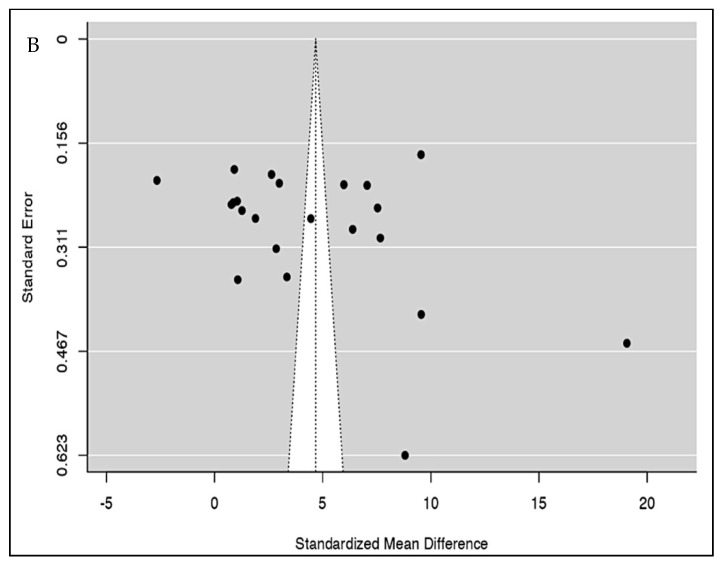
(**A**) Publication bias analysis of all included studies for severity using the funnel plot indicates a potential publication bias. (**B**) Publication bias analysis of all included studies for mortality using the funnel plot indicates a potential publication bias.

Meta-regression analysis was conducted to ascertain the association between NLR values and severity in COVID-19. These were presented as bubble plots. This analysis showed us that the NLR values of patients were not influenced by age (*p* = 0.893), cardiovascular diseases (*p* = 0.259), diabetes mellitus (*p* = 0.545) or hypertension (*p* = 0.104). Both the rank correlation and Egger’s regression tests indicated potential funnel plot asymmetry (*p* = 0.0287 and *p* = 0.0122, respectively) (Figure 4A). However, the trim and fill test did not impute any studies. Leave-one-out analyses demonstrated limited variations in the pooled estimates (max = 2.944, min = 2.737).

### 3.2. Examination of the Relationship between NLR Values and COVID-19 Mortality

**Figure 5:**
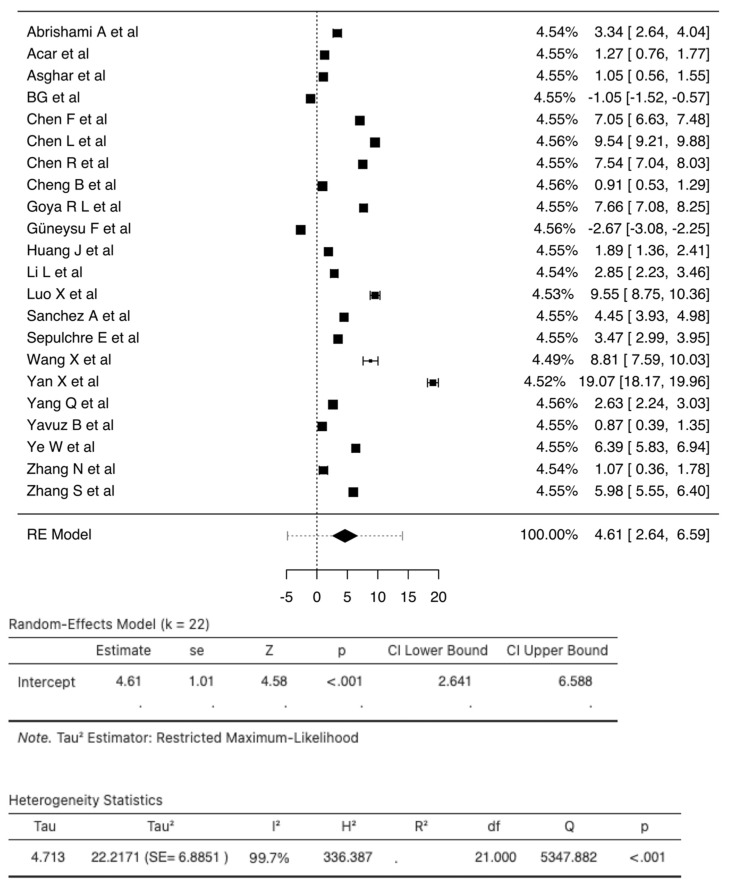
Forest Plot of 22 total studies indicates the pooled SMD calculation that was performed using the Der Simonian–Laird random effect models, observing a value of 4.61 (95% CI: 2.64 to 6.59) between groups. The squares indicate individual effect size while the diamond indicates the summary effect size.

A total of 25 studies consisting of 8351 patients were analyzed in our review. The pooled SMD calculation was conducted using the Der Simonian–Laird random effect models, which observed a value of 4.61 (95% CI: 2.64 to 6.58) between groups with a range from −2.6662 to 19.0655. Even though the Q test showed a high degree of heterogenicity (5347.8816, *p* < 0.0001, tau^2^; = 22.2171, I^2^; = 99.7027%), the 95% prediction interval of the outcomes (ranging from −4.8323 to 14.0613) showed that, although the SMD obtained from the individual studies were generally similar to the pooled SMD estimates, a few studies may have reported a negative true outcome. A study by Yan X et al. [76] showed a large studentized residual value (<± 3.0521), making it a potential outlier. Additionally, analysis of Cook’s distances also showed that the same study had the probability of being overly influential. However, the removal of this study from the analysis did not show a significant difference in the pooled SMD values (3.93 (95% CI: 2.35, 5.50)) (Figure 5). There were three studies [50,63,64] with dichotomized NLR values for which we calculated relative risk. The analysis found that there was an increased risk (RR 2.74; 95% CI 0.98–7.66) of mortality in those with raised NLR when compared to those with normal NLR (Figure S8).

Pooled sensitivity and specificity data were calculated from the 19 studies included for the analysis. The sensitivity estimate was 78.8% (95% CI: 73.5–83.2%), while the specificity estimate was 73.0% (95% CI: 68.4–77.1%). A SROC analysis was carried out, in which AUC was 0.820 while the DOR was 11.483 (95% CI: 7.814–16.875) (Figure 3B and Table 2). The calculation of subgroup analysis at an NLR cut-off value of 6.5 showed that those studied with an NLR cut-off of > 6.5 had significantly higher odds of mortality than those with a cut-off < 6.5 (DOR: 15.5 vs. 7.5) (Table 3). The association between NLR and mortality in COVID-19 was also unaffected by age (*p* = 0.134), cardiovascular diseases (*p* = 0.222), diabetes mellitus (*p* = 0.091), or hypertension (*p* = 0.986) (Figure S7). While checking for publication bias, we found funnel plot asymmetry (*p* = 0.0129) (Figure 4B) but the rank correlation test (*p* = 0.1439) was not significant. Furthermore, trim and fill analysis did not impute any studies.

**Figure 6:**
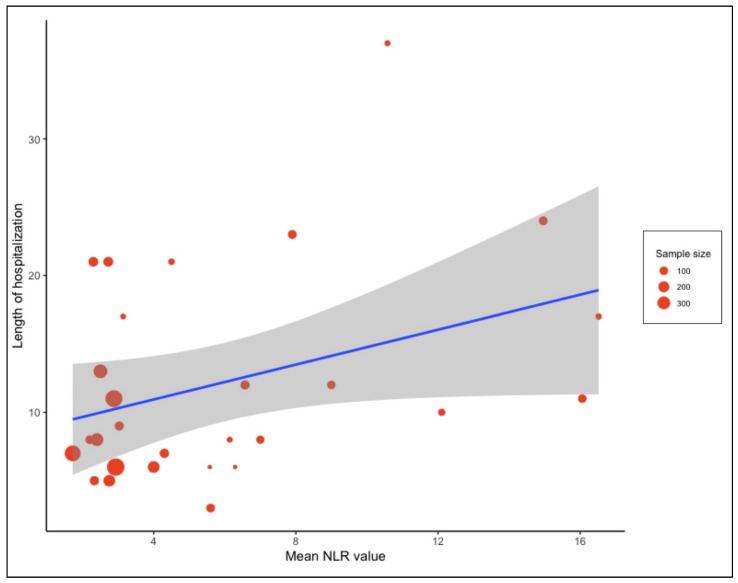
Meta regression analysis presented as bubble plot performed to assess the relationship between the length of hospital stay and NLR value on admission. The blue line indicates the regression line while grey shadow indicate the 95% CI.

Subgroup analysis of COVID-19-associated mortality for NLR estimates according to the different observed WHO regions showed that most of the studies were conducted in the Western Pacific Region (WPR), i.e., n = 13 with 6182 patients. This subgroup had an SMD of 6.39 [4.22, 8.57] and accounted for the highest weight (59%) among the subgroups. A limited number of studies are available from the rest of the world with an SMD ranging from −1.05 [−1.52, −0.57] to 6.39 [4.22, 8.57] (Figure S2). There were minimal differences in the estimated average standardized mean difference between the two subgroups when they were analyzed based on a retrospective and prospective study design (SMD: 4.54 [2.45, 6.63] vs. 4.85 [2.33, 7.38]) (Figure S12). A meta-analysis of the relationship between NLR and length of hospitalization was conducted. It was found that, even though an increased NLR value led to a longer hospital stay, this was not statistically significant (*p* = 0.061) (Figure 6).

## 4. Discussion

In this systematic review, a meta-analysis of sixty-four studies comprising 16,205 patients, we evaluated the role of NLR on admission in the management of COVID-19 patients. Our meta-analysis showed that lower NLR values were correlated with a lower risk of severity and COVID-19-related mortality. NLR was found to be a consistent biomarker for predicting both disease severity (AUC = 0.833, SEN = 80.2% and SPE = 75.8%) and mortality (AUC = 0.820, SEN = 78.8% and SPE = 73.0%) in COVID-19. A novelty of this study was subgroup classification based on severity criteria and region, both of which did not yield statistically significant differences between subgroups. We also found no significant relationship between the length of hospitalization and NLR values on admission. Our study also showed that the relationship between NLR and COVID-19 outcomes was independent of age and comorbidities such as diabetes mellitus, cardiovascular diseases, and hypertension.

The severity and mortality of COVID-19 is correlated with the extensive infiltration of neutrophils in the lung and neutrophil numbers in the peripheral blood, and the magnitude of neutrophilia is suggestive of the intensity of inflammatory responses [86]. Additionally, studies have shown that COVID-19 primarily affects CD4+ T and CD8+ T cells. We see that the development of lymphopenia is largely due to its diminished CD8+ T count during the first week of the disease [87].

NLR was first proposed as a prognostic marker in critically ill patients, as it correlated well with Acute Physiology and Chronic Health Evaluation (APACHE II) scoring and Sequential Organ Failure Assessment score (SOFA score) [88]. It is a marker of systemic inflammation, which is used in conditions such as tumors, pancreatitis, chronic obstructive pulmonary disease (COPD), and cardiovascular disease. It has also been used in the prognosis of infectious diseases, such as influenza virus infection and Middle East respiratory syndrome (MERS) [11,12].

Apart from the criteria used for COVID-19 disease stratification, there are other clinical scoring systems, which include the most widely used APACHE II scoring, COVID-19 Critical Illness Prediction Tool (COVID-GRAM), SOFA score and Comorbidity, Age, Lymphocyte Count, Lactate Dehydrogenase score (CALL score) [89]. However, the drawback of some of these clinical scoring systems, such as the APACHE II and COVID-GRAM, is that they have more than ten variables, some of which rely on advanced laboratory tests such as lactate dehydrogenase (LDH), serum electrolytes, and arterial pH. Other biomarkers, such as interleukin-6, D-dimer levels, C-reactive protein (CRP) and Soluble urokinase plasminogen activator receptor (suPAR), are expensive and inaccessible, which makes it difficult to use in healthcare settings in Low and Low–Middle Income Countries (LICs/LMICs) with constrained capacity and resources. Hence, simpler tools for predicting COVID-19 severity and mortality, such as NLR, have the advantage of having a quicker turn-around and being inexpensive [90,91,92].

At the time of writing this manuscript, a few other reviews have evaluated NLR as a prognostic indicator [13,14,15,16]. This review includes almost twice the number of studies compared to other published reviews, which increased the comprehensiveness of the review. All the other reviews used only oxygenation-based severity criteria, while our review used different severity criteria, and we performed subgroup analysis to analyze SMD in the various criteria used. Criteria based on the need for IMV and respiratory rate and oxygenation showed the closest approximation to the total estimate (Figure S1A).

The standardized mean difference in NLR values was calculated in only one other review [14], which we observed to occur in the same direction as our study. However, we observed that the NLR values that best predicted severity and mortality outcomes were higher in our study. This may be because our analysis incorporated different severity criteria and included a larger number of studies. There is no consensus on the optimal ‘cut-off’ value for NLR, to predict clinically relevant outcomes, especially for COVID-19. In determining the optimal ‘cut-off’ value, this review of 21 studies that identified cut-off values for NLR showed a wide range, between 2.306 [69] and 13.4 [65], in predicting severity, and 19 studies observed NLR values ranging from 3.2 [30] to 12.0 [55] for predicting mortality. There may be an ethnic-demographical element to these inconsistencies, and NLR is known to be affected by them [17]. We need more studies analyzing these cut-off values and their relationship with these population subgroups.

A region-wise stratification of studies reporting NLR for outcomes is a novelty to this review. One region (Western Pacific Region) contributed the largest percentage of studies (Figures S1B and S2). We found a large variation between the various WHO regions in terms of both severity and mortality outcomes. Previous studies have demonstrated that NLR values are also determined by race and ethnicity [17]. Additionally, none of the studies have adjusted for the confounding effects of factors such as tobacco smoking, which are known to influence NLR values [93]. We need a greater number of studies from different WHO regions to further investigate these findings.

We found that the sensitivity and specificity analysis performed by three other systematic reviews was in close approximation to our review, with an AUC ranging from 0.81 to 87, with similar NLR cut-off ranges (mortality from 6 to 6.5 and severity from 4 to 4.5) [13,15,16]. Interestingly, similar to two other reviews, our analysis also found that a higher cut-off value of NLR (>6.5) had more than twice the odds ratio than those with a low cut-off value (<6.5) when predicting mortality [13,16].

NLR values have been reported to vary with age, sex, and underlying comorbid conditions, such as diabetes mellitus, cardiovascular diseases, and hypertension [17,94,95,96]. Although most studies in the systematic review showed a statistically significant difference in the aforementioned factors between the groups, a meta-regression analysis illustrated that the association between the severity and mortality of COVID-19 disease and NLR was independent of age, sex, and underlying comorbid conditions, such as diabetes mellitus, cardiovascular diseases, and hypertension. A similar observation was reported in two relatively smaller systematic reviews by Simadibrata et al. and Kumar et al. [13,14].

There are a few limitations to the currently available evidence. First, most of the included studies were conducted in a single country, i.e., China. Second, data were mostly from retrospective studies, which were prone to confounding factors. Third, even though trim and fill analysis did not impute any studies, we found that there was a significant publication bias in studies assessing both severity and mortality. Fourth, there is no information on whether this patient was previously admitted to other hospitals, and there was limited information on the time difference between the onset of syndromes and the NLR sample being taken. Furthermore, no data were available on vaccination status, treatment at the time of hospital admission, or confounders such as smoking. The comorbidities assessed in most of the studies were limited to only a few major conditions. Conditions such as obesity, chronic pulmonary disease and variations associated with anti-inflammatory/immunomodulating therapy were not assessed. Finally, there was considerable heterogeneity among the included studies, which was not identifiable.

The COVID-19 pandemic is currently in its third year. There are various vaccines available for the disease, and the vaccination rates greatly differ from place to place [97,98,99]. Vaccination alters immune responses in COVID-19 patients, which may hamper the utility and/or validity of using the NLR. However, a study performed by Mediu et al., which evaluated NLR values in vaccinated patients, showed that there was no statistical significance between cases and controls [100]. In the future, we need further large-scale, prospective studies to clinically validate a more exact NLR cut-off. There is a need to conduct these studies in various other demographics to assess ethnic and racial differences. We would also need to account and adjust for the date of NLR collection and the duration of symptoms, treatment at the time of hospitalization and other potential confounders. Lastly, the pandemic has also seen a shift in the clinical evaluation of patients with the onset of telemedicine and remote counselling [101]. Thus, there is a need to also assess the best markers to accommodate the changing healthcare scenario.

## 5. Conclusions

NLR is a useful assessment tool to map out COVID-19-related disease severity and mortality outcomes. Embedding NLR into routine clinical management can help clinicians to identify potentially severe cases earlier, and facilitate risk stratification to initiate prompt therapeutic intervention. We observed that NLR > 6.5 is associated with significantly greater the odds of mortality. There is a need for further studies that focus on obtaining a clinically relevant cut-off value that may potentially improve clinical outcomes and reduce overall COVID-19-related mortality.

## Figures and Tables

**Table 1:** Table depicting the baseline characteristics of the included studies.

Sl. No.	Study	Country	Study Design	Year	N	Outcome Measured	NOS Score
1	Abrishami A et al. [22]	Iran	Prospective	2021	100	Mortality	7
						ROC analysis	
2	Acar et al. [23]	Turkey	Prospective	2021	148	Mortality	7
						ROC analysis	
3	Asghar et al. [24]	Pakistan	Retrospective	2020	100	Severity	7
						Mortality	
						ROC analysis	
4	Bastug A et al. [25]	Turkey	Retrospective	2020	191	Severity	7
						ROC analysis	
5	BG et al. [26]	India	Retrospective	2021	100	Mortality	7
						ROC analysis	
6	Chen F et al. [27]	China	Retrospective	2020	681	Mortality	7
						ROC analysis	
7	Chen L et al. [28]	China	Prospective	2020	1859	Mortality	9
8	Chen R et al. [29]	China	Retrospective	2020	548	Severity	9
						Mortality	
9	Cheng B et al. [30]	China	Retrospective	2020	456	severity	8
						Mortality	
						ROC analysis	
10	Ding X et al. [31]	China	Retrospective	2020	72	Severity	8
11	Fei M et al. [32]	China	Retrospective	2020	72	Severity	5
						ROC analysis	
12	Fu J et al. [33]	China	Retrospective	2020	75	Severity	6
						ROC analysis	
13	Ghazanfari T et al. [34]	Turkey	Prospective	2021	93	ROC analysis	7
14	Gong J et al. [35]	China	Retrospective	2020	372	Severity	7
						ROC analysis	
15	Goya R L et al. [36]	Spain	Prospective	2020	501	Mortality	6
						ROC analysis	
16	Guner R et al. [37]	Turkey	Prospective	2020	222	Severity	6
17	Güneysu F et al. [38]	Turkey	Retrospective	2020	169	Mortality	7
						ROC analysis	
18	Hammad R et al. [39]	Egypt	Prospective	2021	64	Severity	7
						ROC analysis	
19	Hu H et al. [40]	China	Retrospective	2020	40	Severity	6
						ROC analysis	
20	Huang J et al. [41]	China	Retrospective	2020	299	Mortality	8
21	Kazancioglu S et al. [42]	China	Retrospective	2020	181	Severity	8
22	Kong M et al. [43]	China	Retrospective	2020	210	Severity	7
23	Li L et al. [44]	China	Retrospective	2020	93	Mortality	7
24	Liao D et al. [45]	China	Retrospective	2020	466	Severity	7
25	Lin S et al. [46]	China	Retrospective	2021	68	Severity	7
						ROC analysis	
26	Liu F et al. [47]	China	Retrospective	2020	134	Severity	8
						ROC analysis	
27	Liu J et al. [48]	China	Prospective	2020	115	Severity	7
						ROC analysis	
28	Liu YP et al. [49]	China	Retrospective	2020	84	Severity	8
						ROC analysis	
29	Liu Y [50]	China	Retrospective	2020	245	Mortality	7
30	Luo X et al. [51]	China	Retrospective	2020	298	Mortality	8
						ROC analysis	
31	Ok F et al. [52]	Turkey	Prospective	2021	139	Severity	7
						ROC analysis	
32	Qin C et al. [53]	China	Retrospective	2020	452	Severity	5
33	Ramesh J et al. [54]	India	Retrospective	2021	154	ROC analysis	8
34	Sanchez A et al. [55]	Mexico	Prospective	2020	242	Mortality	6
						ROC analysis	
35	Sayah W et al. [56]	Algeria	Prospective	2021	153	Severity	8
						ROC analysis	
36	Sayed A et al. [57]	Saudi Arabia	Retrospective	2021	951	Severity	7
						ROC analysis	
37	Seo J et al. [58]	Korea	Retrospective	2021	166	ROC analysis	7
38	Sepulchre E et al. [59]	Belgium	Retrospective	2020	198	Severity	7
						Mortality	
						ROC analysis	
39	Shang W et al. [60]	China	Retrospective	2020	443	Severity	7
						ROC analysis	
40	Shi S et al. [61]	China	Prospective	2021	87	Severity	6
						ROC analysis	
41	Sun S et al. [62]	China	Prospective	2020	116	Severity	5
						ROC analysis	
42	Tatum et al. [63]	USA	Prospective	2020	125	Mortality	6
						ROC analysis	
43	Ullah [64]	USA	Retrospective	2020	176	Mortality	6
44	Wang C et al. [65]	China	Retrospective	2020	45	Severity	7
						ROC analysis	
45	Wang F et al. [66]	China	Retrospective	2020	333	Severity	8
46	Wang K et al. [67]	China	Retrospective	2021	38	Severity	7
						ROC analysis	
47	Wang W et al. [68]	China	Retrospective	2020	123	Severity	7
						ROC analysis	
48	Wang X et al. [69]	China	Retrospective	2020	131	Mortality	7
						Severity	
						ROC analysis	
49	Wu S et al. [70]	China	Retrospective	2020	270	Severity	7
						ROC analysis	
50	Xia X et al. [71]	China	Retrospective	2020	63	Severity	8
						ROC analysis	
51	Xie G et al. [72]	China	Retrospective	2020	324	Severity	
						ROC analysis	5
52	Xie L et al. [73]	China	Retrospective	2020	373	Severity	5
53	Xu J et al. [74]	China	Retrospective	2020	76	ROC analysis	5
54	Xue G et al. [75]	China	Retrospective	2020	114	Severity	7
						ROC analysis	
55	Yan X et al. [76]	China	Retrospective	2020	1004	Mortality	8
						ROC analysis	
56	Yang AP et al. [77]	China	Retrospective	2020	93	Severity	7
						ROC analysis	
57	Yang Q et al. [78]	China	Retrospective	2020	226	Mortality	8
58	Yavuz B et al. [79]	Turkey	Retrospective	2021	113	Mortality	9
						ROC analysis	
59	Ye W et al. [80]	China	Retrospective	2020	349	Mortality	8
						ROC analysis	
60	Zhang N et al. [81]	China	Retrospective	2020	60	Mortality	6
61	Zhang S et al. [82]	China	Retrospective	2020	115	Mortality	7
62	Zhang Y et al. [83]	China	Retrospective	2020	115	Severity	7
63	Zhou Y et al. [84]	China	Retrospective	2020	442	Severity	7
64	Zhu Z et al. [85]	China	Retrospective	2020	127	Severity	5

**Table 2:** Sensitivity, specificity, AUC and DOR analyses of NLR for predicting disease severity and mortality in patients with COVID-19.

Categories	No. of Studies	*p*-Value	Estimates	AUC	DOR
NLR for predicting disease mortality					
Sensitivity	19	0.013	78.8% (95% CI: 73.5–83.2)	0.820	11.483
Specificity		<0.001	73.0% (95% CI: 68.4–77.1)		
NLR for predicting disease severity					
Sensitivity	21	<0.001	80.2% (95% CI: 74.0–85.2)	0.833	13.63
Specificity		<0.001	75.8% (95% CI 71.3–79.9)		

**Table 3:** Subgroup analysis of NLR cut-offs for COVID-19 severity and mortality.

Categories	No. of Studies	Sensitivity	Specificity	AUC	OR
**Severity**					
Subgroup A (NLR cut off < 4.5)	13	81.9%	74.1%	0.834	13.032
Subgroup B (NLR cut off > 4.5)	8	80.0%	75.9%	0.833	13.511
**Mortality**					
Subgroup A (NLR cut off < 6.5)	10	79.8%	65.6%	0.800	7.585
Subgroup B (NLR cut off > 6.5)	9	78.6%	73.4%	0.854	15.581

## Data Availability

All the data generated or analyzed during this study are included in this published article and are available from the corresponding author upon reasonable request. This review was registered on the PROSPERO database (CRD42021252100).

## Supplementary Materials

The following supporting information can be downloaded at: https://www.mdpi.com/article/10.3390/vaccines10081233/s1, Table S1: Prisma Checklist, Table S2: Search strategy terms and results, Table S3: Identification of various severity criteria for studies, Table S4: The characteristics of the included studies comparing severe and non-severe COVID-19 patients, Table S5: The characteristics of included studies, comparing survivors and non-survivors of COVID-19, Table S6: Risk of bias assessment of all included studies using the Newcastle–Ottawa Scale (NOS), Figure S1A: Forest plot of studies comparing severe disease with non-severe disease, stratified by severity criteria, Figure S1B: Forest plot of studies comparing severe disease with non-severe disease, stratified by WHO region, Figure S2: Forest plot of studies comparing non-survivors with survivors, stratified by WHO region, Figure S3A: Forest plot of the NLR odds for predicting disease severity in patients with COVID-19, Figure S3B: Forest plot of NLR odds for predicting disease mortality in patients with COVID-19, Figure S4A: Forest plot showing NLR’s sensitivity for predicting disease severity in patients with COVID-19, Figure S4B: Forest plot showing NLR’s specificity for predicting disease severity in patients with COVID-19, Figure S5: Forest plot showing diagnostic odds ratio (DOR) of NLR for predicting disease severity in patients with COVID-19, Figure S6: Forest plot showing sensitivity and specificity of NLR for predicting disease mortality in patients with COVID-19, Figure S7: Forest plot showing sensitivity and specificity of NLR for predicting disease mortality in patients with COVID-19, Figure S8: Forest Plot using the random-effects model, showing the association between NLR value on admission and all-cause mortality risk, Figure S9: Bubble plot for meta-regression in studies with severity outcome, Figure S10: Bubble plot for meta-regression in studies with mortality outcome, Figure S11: Forest plot of studies comparing severe disease with non-severe disease, stratified by study design, Figure S12: Forest plot of studies comparing non-survivors with survivors, stratified by region.
