# Supplementary material for: The Role of Neutrophil-to-Lymphocyte Ratio in Risk Stratification and Prognostication of COVID-19: A Systematic Review and Meta-Analysis
Source: Vaccines (Basel). 2022 Aug 1;10(8):1233. doi: 10.3390/vaccines10081233 (PMC9415708; doi:10.3390/vaccines10081233)

vaccines

MDPI

Systematic Review and Meta-Analysis

Supplementary Files : The role of Neutrophil-to-Lymphocyte ratio in Risk Stratification and Prognostication of COVID-19

Ashwaghosha Parthasarathi[1], Sunag Padukudru[2], Sumalata Arunachal[3], Chetak Kadabasal Basavaraj[3], Mamidipudi Thirumala Krishna[4], Koustav Ganguly[5], Swapna Upadhyay[5*], Padukudru Anand Mahesh[3*]

1. Allergy, Asthma, and Chest Centre, Krishnamurthypuram, Mysore, Karnataka, India
2. Yenepoya Medical College, Yenepoya University, Mangalore, Karnataka, India
3. Department of Respiratory Medicine, JSS Medical College, JSSAHER, Mysore, Karnataka, India
4. University Hospitals Birmingham NHS Foundation Trust; Institute of Immunology Immunotherapy, University of Birmingham, UK
5. Unit of Integrative Toxicology, Institute of Environmental Medicine (IMM), Karolinska Institutet, 17177 Stockholm, Sweden

* equal contribution and corresponding authors

* Correspondence: PAM : **mahesh1971in@yahoo.com; SU: swapna.upadhyay@ki.se**

List of Supplementary Files

Table S1. PRISMA checklist

Table S2. Search strategy for peer-reviewed databases

Table S3. The characteristics of included studies comparing severe and non-severe COVID-19 patients

Table S4. The characteristics of included studies comparing survivors and non-survivors of COVID-19

Table S5. Risk of bias assessment of all included studies using the Newcastle-Ottawa Scale (NOS)

Figure S1 A: Forest plot of studies comparing severe disease with non-severe disease, stratified by severity criteria

Figure S1 B: Forest plot of studies comparing severe disease with non-severe disease, stratified by region

Figure S2: Forest plot of studies comparing non-survivors with survivors, stratified by region

Figure S3 A: Forest plot of the odds of NLR for predicting disease severity in patients with COVID-19.

Figure S3 B: Forest plot of odds of NLR for predicting disease mortality in patients with COVID-19.

Figure S4 A: Forest plot showing sensitivity of NLR for predicting disease severity in patients with COVID-19.

Figure S4 B: Forest plot showing specificity of NLR for predicting disease severity in patients with COVID-19.

Figure S5: Forest plot showing diagnostic odds ratio (DOR) of NLR for predicting disease severity in patients with COVID-19.

Figure S6: Forest plot showing sensitivity and specificity of NLR for predicting disease Mortality in patients with COVID-19.

Figure S7: Forest plot showing sensitivity and specificity of NLR for predicting disease Mortality in patients with COVID-19.

Figure S8: Forest Plot using the random-effects model showing the association between NLR value on admission and all-cause mortality risk.

Figure S9: Bubble plot for meta-regression in studies with severity outcome.

Figure S10: Bubble plot for meta-regression in studies with mortality outcome

Figure S11: Forest plot of studies comparing severe disease with non-severe disease stratified by study design

Figure S12 : Forest plot of studies comparing non-survivors with survivors, stratified by study design

Table S1: Prisma Checklist

Section and Topic	Item #	Checklist item	Location where item is reported
**TITLE**			
Title	1	Identify the report as a systematic review.	Page 1
**ABSTRACT**			
Abstract	2	See the PRISMA 2020 for Abstracts checklist.	
**INTRODUCTION**			
Rationale	3	Describe the rationale for the review in the context of existing knowledge.	Page 2
Objectives	4	Provide an explicit statement of the objective(s) or question(s) the review addresses.	Page 2
**METHODS**			
Eligibility criteria	5	Specify the inclusion and exclusion criteria for the review and how studies were grouped for the syntheses.	Page 3
Information sources	6	Specify all databases, registers, websites, organisations, reference lists and other sources searched or consulted to identify studies. Specify the date when each source was last searched or consulted.	Page 3
Search strategy	7	Present the full search strategies for all databases, registers and websites, including any filters and limits used.	Page 3
Selection process	8	Specify the methods used to decide whether a study met the inclusion criteria of the review, including how many reviewers screened each record and each report retrieved, whether they worked independently, and if applicable, details of automation tools used in the process.	Page 3,4
Data collection process	9	Specify the methods used to collect data from reports, including how many reviewers collected data from each report, whether they worked independently, any processes for obtaining or confirming data from study investigators, and if applicable, details of automation tools used in the process.	Page 3,4

Section and Topic	Item #	Checklist item	Location where item is reported
Data items	10a	List and define all outcomes for which data were sought. Specify whether all results that were compatible with each outcome domain in each study were sought (e.g. for all measures, time points, analyses), and if not, the methods used to decide which results to collect.	Page 3,4
	10b	List and define all other variables for which data were sought (e.g. participant and intervention characteristics, funding sources). Describe any assumptions made about any missing or unclear information.	-
Study risk of bias assessment	11	Specify the methods used to assess risk of bias in the included studies, including details of the tool(s) used, how many reviewers assessed each study and whether they worked independently, and if applicable, details of automation tools used in the process.	Page 4
Effect measures	12	Specify for each outcome the effect measure(s) (e.g. risk ratio, mean difference) used in the synthesis or presentation of results.	Page 4,5
Synthesis methods	13a	Describe the processes used to decide which studies were eligible for each synthesis (e.g. tabulating the study intervention characteristics and comparing against the planned groups for each synthesis (item #5)).	Figure 1
	13b	Describe any methods required to prepare the data for presentation or synthesis, such as handling of missing summary statistics, or data conversions.	-
	13c	Describe any methods used to tabulate or visually display results of individual studies and syntheses.	Page 4
	13d	Describe any methods used to synthesize results and provide a rationale for the choice(s). If meta-analysis was performed, describe the model(s), method(s) to identify the presence and extent of statistical heterogeneity, and software package(s) used.	Page 4,5
	13e	Describe any methods used to explore possible causes of heterogeneity among study results (e.g. subgroup analysis, meta-regression).	Page 5
	13f	Describe any sensitivity analyses conducted to assess robustness of the synthesized results.	-
Reporting bias assessment	14	Describe any methods used to assess risk of bias due to missing results in a synthesis (arising from reporting biases).	-
Certainty assessment	15	Describe any methods used to assess certainty (or confidence) in the body of evidence for an outcome.	-
**RESULTS**			

Section and Topic	Item #	Checklist item	Location where item is reported
Study selection	16a	Describe the results of the search and selection process, from the number of records identified in the search to the number of studies included in the review, ideally using a flow diagram.	Page 5
	16b	Cite studies that might appear to meet the inclusion criteria, but which were excluded, and explain why they were excluded.	Page 5
Study characteristics	17	Cite each included study and present its characteristics.	Table 1
Risk of bias in studies	18	Present assessments of risk of bias for each included study.	-
Results of individual studies	19	For all outcomes, present, for each study: (a) summary statistics for each group (where appropriate) and (b) an effect estimate and its precision (e.g. confidence/credible interval), ideally using structured tables or plots.	Table number
Results of syntheses	20a	For each synthesis, briefly summarise the characteristics and risk of bias among contributing studies.	-
	20b	Present results of all statistical syntheses conducted. If meta-analysis was done, present for each the summary estimate and its precision (e.g. confidence/credible interval) and measures of statistical heterogeneity. If comparing groups, describe the direction of the effect.	Page 6-8
	20c	Present results of all investigations of possible causes of heterogeneity among study results.	-
	20d	Present results of all sensitivity analyses conducted to assess the robustness of the synthesized results.	-
Reporting biases	21	Present assessments of risk of bias due to missing results (arising from reporting biases) for each synthesis assessed.	-
Certainty of evidence	22	Present assessments of certainty (or confidence) in the body of evidence for each outcome assessed.	-
**DISCUSSION**			
Discussion	23a	Provide a general interpretation of the results in the context of other evidence.	Page 8-11

Section and Topic	Item #	Checklist item	Location where item is reported
	23b	Discuss any limitations of the evidence included in the review.	Page 11
	23c	Discuss any limitations of the review processes used.	-
	23d	Discuss implications of the results for practice, policy, and future research.	-
**OTHER INFORMATION**			
Registration and protocol	24a	Provide registration information for the review, including register name and registration number, or state that the review was not registered.	
	24b	Indicate where the review protocol can be accessed, or state that a protocol was not prepared.	
	24c	Describe and explain any amendments to information provided at registration or in the protocol.	
Support	25	Describe sources of financial or non-financial support for the review, and the role of the funders or sponsors in the review.	
Competing interests	26	Declare any competing interests of review authors.	
Availability of data, code and other materials	27	Report which of the following are publicly available and where they can be found: template data collection forms; data extracted from included studies; data used for all analyses; analytic code; any other materials used in the review.	

From: Page MJ, McKenzie JE, Bossuyt PM, Boutron I, Hoffmann TC, Mulrow CD, et al. The PRISMA 2020 statement: an updated guideline for reporting systematic reviews. BMJ 2021;372:n71. doi: 10.1136/bmj.n71

For more information, visit: http://www.prisma-statement.org/

Table S2: Search strategy terms and results:

Database	Keywords
PubMed, Medline and Embase	("coronavirus disease 2019"[Title/Abstract] OR "2019 novel coronavirus"[Title/Abstract] OR "SARS-CoV-2"[Title/Abstract] OR "2019-nCoV"[Title/Abstract] OR "COVID-19"[Title/Abstract]) AND (("neutrophil to lymphocyte ratio"[Title/Abstract] OR "neutrophil lymphocyte ratio"[Title/Abstract] OR "neutrophil to lymphocyte ratio"[Title/Abstract]) AND "neutrophil lymphocyte ratio"[Title/Abstract] AND "NLR"[Title/Abstract])
Scopus	(TITLE-ABS-KEY(COVID-19) OR TITLE-ABS-KEY(COVID19) OR TITLE-ABS-KEY(COVID) OR TITLE-ABS-KEY("coronavirus disease 2019") OR TITLE-ABS-KEY(2019-ncov) OR TITLE-ABS-KEY(sars-cov-2)) AND (TITLE-ABS-KEY(neutrophil-to-lymphocyte)

	OR TITLE-ABS-KEY(NLR)) AND (TITLE-ABS-KEY(sever*) OR TITLE-ABS-KEY(“intensive care unit”) OR TITLE-ABS-KEY(ICU) OR TITLE-ABS-KEY(mortality) OR TITLE-ABS-KEY(death) OR TITLE-ABS-KEY(non-survivor))
Cochrane library	("Covid-19" OR "COVID19" OR "COVID" OR "coronavirus disease 2019" OR "2019-nCoV" OR "SARS-CoV-2") in Title Abstract Keyword AND (neutrophil-to-lymphocyte OR NLR) in Title Abstract Keyword AND (sever* OR "intensive care unit" OR ICU OR mortality OR death OR non-survivor)

Table S3: Identification of various severity criteria for studies			
**Sl. No.**	**Study**	**Severity criteria**	**Non-severe criteria**
1	Asghar et al (43)	ICU admitted patients were considered as having severe disease. Those not admitted to ICU were not considered severe	Those not admitted to ICU were not considered non-severe.
2	Bastug A et al (77)	Patients requiring treatment in an intensive care unit (ICU) on admission or at some point during hospital stay (ICU group) are considered as having severe disease.	Those not admitted to ICU were not considered severe.
3	Chen R et al (79)	The severity of the disease was assessed according to the Seventh Version of the Novel Coronavirus Pneumonia Diagnosis and Treatment Guidance from the National Health Commission of China.	Those not were not considered severe.
4	Cheng B et al (62)	Severe grade was defined as respiratory frequency of 30 breaths/min, blood oxygen saturation of 93%, oxygenation index <300 mmHg and/or lung infiltrates >50% within 24-48 hours.	All other patients were non-severe
5	Ding X et al (80)	Patients whose imaging showed that the pulmonary involvement significantly aggravated by more than 50% within 24–48 h were managed as severe type	All other patients were non-severe
6	Fei M et al (44)	Patients whose imaging showed that the pulmonary involvement significantly aggravated by more than 50% within 24–48 h were managed as severe type	All other patients were non-severe
7	Fu J et al (81)	The severe group consisted of severe and critical cases which fulfilled one of these criteria as follows: 1) respiratory distress (RR ≥ 30 bpm); 2) oxygen saturation ≤ 93%; 3) arterial partial pressure of oxygen (PaO2)/fraction of inspired oxygen (FiO2) < 300 mm Hg; 4) patients with chest imaging that indicates an obvious progression of infiltrations within 24–48 h; 5) respiratory failure and requiring mechanical ventilation, shock or other organ failure need ICU support	All other patients were non-severe
8	Gong J et al (82)	Not Mentioned	

9	Guner R et al (83)	Severe acute respiratory illness (SARI)/ critical disease according to WHO guidelines	
10	Hammad R et al (47)	Severe patients additionally met at least one of the following conditions: (a) Shortness of breath, respiration rate ≥ 30 times/min, (b) oxygen saturation (resting state) ≤ 93%	Non-severe patients met all following conditions: (a) history of exposure to a confirmed SARS-CoV-2 patient, (b) fever or other respiratory symptoms, and (c) typical chest computed tomography image abnormities compatible with viral pneumonia.
11	Hu H et al (48)	Not Mentioned	
13	Kazancioglu S et al(86)	The severe illness was defined as:<br>1. Respiratory frequency >30 breaths per min,<br>2. SpO2 <94% on room air at sea level, a ratio of the arterial partial pressure of oxygen to fraction of inspired oxygen (PaO2/FiO2) <300 or lung infiltrates >50%.	All other patients were non-severe
14	Kong M et al (87)	The condition was considered as severe-type COVID-19 when one of the following criteria was present:<br>(1) Respiratory distress with respiratory rate >30/min;<br>(2) oxygen saturation ≤93% in the resting state; or<br>(3) arterial blood oxygen partial pressure (PaO2)/oxygen concentration (FiO2) ≤300 mmHg (1 mmHg = 0.133 kPa).	All other patients were non-severe
15	Liao D et al (89)	Severe cases were defined as respiratory distress (≥30 breaths per min), oxygen saturation of 93% or less at rest, ratio of arterial partial pressure of oxygen to fractional concentration of oxygen in inspired air of 40 kPa or less, or more than 50% lesion progression over 24–48 h in pulmonary imaging.	Moderate disease was defined as fever and respiratory symptoms with radiological findings of pneumonia but without the severe or critical features. Patients with mild disease, defined as mild clinical symptoms and no sign of pneumonia on imaging, did not require hospital admission so were not included in this study.
16	Lin S et al(90)	Severe disease: respiratory failure requiring mechanical ventilation, shock, and/or combined organ failure requiring admission to the intensive care unit.	Mild disease: fever, respiratory symptoms, and imaging findings suggestive of pneumonia;<br>moderate disease: respiratory distress with a respiratory rate of ≥30 breaths/min, mean oxygen saturation of ≤93% in a resting state, and/or arterial blood oxygen partial pressure of ≤300mmHg

17	Liu F et al (91)	Severe illness was defined as a condition with any severe event based on one major criterion, two or more minor criteria, or two criteria of additional organ dysfunction, as follows:<br>(a) Major criteria: respiratory failure requiring mechanical ventilation; shock with the need for vasopressors; extracorporeal membrane oxygenation (ECMO) treatment;<br>(b) Minor criteria: multilobar infiltrates; respiratory rate ≥ 30 breaths/min; arterial oxygen pressure (PaO2) < 60 mmHg; PaO2/FiO2 ratio ≤ 300 mmHg; oxygen saturation ≤ 93%; hemoptysis 24 h ≥ 100 mL;	All other patients were non-severe
18	Liu J et al (50)	Severe: met any of the following: (a) respiratory distress, respiratory rate ≥ 30 beats/min; (b) in the resting state, mean oxygen saturation ≤ 93%; (c) arterial blood oxygen partial pressure/oxygen concentration ≤ 300 mmHg (1 mmHg = 0.133 kPa).<br>(4) critical, meets any of the following: (a) respiratory failure occurs and requires mechanical ventilation; (b) Shock occurs; (c) ICU admission is required for combined organ failure.	All other patients were non-severe
19	Liu Y et al (51)	Severe: patient who meets any of the following conditions: patients present respiratory distress with respiratory rate ≥30 breath/min, SpO2 (oxygen saturation) ≤93% on room air, and PaO2 (arterial blood oxygen partial pressure)/FiO2 (fraction of inspired oxygen) ≤300 mmHg (1 mmHg =0.133 kPa);<br>Critical: patient meets any of the following conditions: patient presents respiratory failure and requires mechanical ventilation support, patient presents shock, and patient presents multiple organ dysfunction syndrome and requires ICU admission	mild: patients present mild symptoms with no imaging sign of pneumonia;<br>common: patients have fever and respiratory tract symptom with imaging sign of pneumonia
20	Ok F et al (45)	Severe patients also had at least one of the following features; PaO2/FiO2 ≤300 mm Hg, or oxygen saturation (at rest) less than 93%, or shortness of breath, respiratory rate ≥30 times/minute.	Nonsevere patients met all of the features such as the history of contact, respiratory symptoms or fever, typical viral pneumonia involvement in computed tomography, and positive test result of RT-PCR for SARS-CoV-2 RNA.
21	Qin C et al (93)	Those who met the following criteria were defined as having severe-type infection: (1) respiratory distress with a respiratory rate over 30 breaths per minute, (2) oxygen saturation ≤93% in the resting state, and (3) arterial blood oxygen partial pressure (PaO2) /oxygen concentration (FiO2) ≤300 mm Hg.	All other patients were non-severe
22	Sayah W et al (52)	The patients were classified based on the severity of symptoms according to the World Health Organization (WHO) interim guidance for COVID-19	All other patients were non-severe

23	Sayed A et al (58)	Intensive care unit admission criteria were as follows: a temperature of 38.5°C accompanied with a decrease in mean arterial pressure below 60 mmHg or shortness of breath that is New York Heart Association (NYHA) class III or higher which is characterized as "marked limitation in activity due to symptoms, even during less-than-ordinary activity, example: walking short distances (20-100 m). Comfortable only at rest.	confirmed cases by qPCR, symptomatic, low oxygen saturation <94% on room air and clinical or radiological evidence of pneumonia.
24	Seo J et al (53)	Oxygen was supplied to patients with oxygen saturation less than 93% in room air. The definition of ARDS is a partial pressure of arterial oxygen (PaO_2)/percentage of inspired oxygen (FiO_2) of <300 mmHg	Pulmonary infiltration was classified as patchy, confluent, or nodular, and unilateral or bilateral, by at least two physicians in each hospital. The area of pulmonary infiltration was analyzed by using DEEP:PHI (medical AI software; DEEPNOID, Seoul, Republic of Korea) which is an open platform that supports medical imaging artificial intelligence (AI) model research efficiently.
25	Sepulchre E et al (54)	The unfavorable evolution (UFE) group included patients admitted to intensive care unit (ICU) and/or patients who died during hospitalization	the favorable evolution (FE) group included the non-ICU surviving patients.
26	Shang W et al (55)	The intensive and critical types were classified as a severe group.<br>Intensive type met any of the following: shortness of breath, respiratory rate more than 30 times/min; resting state, oxygen saturation less than equal to 93%; or partial pressure of arterial oxygen (PaO2)/oxygen concentration (FiO2) ≤ 30 mm Hg (1 mm Hg = 0.133 kPa); the critical type was defined as respiratory failure requiring mechanical ventilation, shock, or other organ failure requiring ICU monitoring and treatment.	The light and ordinary types were classified as the non-severe group<br>Light type: no pneumonia on imaging; Ordinary type: fever and respiratory tract symptoms with pneumonia in imaging;
27	Shi S et al (56)	An adverse prognosis was considered as the admission to the intensive care unit (ICU) or death.<br>He criterion for admission to the ICU was either (a) respiratory failure occurrence that requires mechanical ventilation;<br>(b)Shock occurrence; or<br>(c)Combined with other organ failure requiring ICU monitoring and treatment.	All other patients were non-severe
28	Sun S et al (57)	Severe cases: Those who have one of the following three clinical manifestations: (1). shortness of breath with RR > 30 times/min; (2). mean oxygen saturation ≤ 93% in resting state; (3). partial pressure of arterial oxygen (PaO2)/oxygen Concentration (FiO2) ≤ 300 mmHg (1 mmHg = 0.133 kPa).	Common cases: Those who have fever, respiratory tract symptoms, and pneumonia on imaging.

29	Wang C et al (42)	Meanwhile, in addition to having the symptoms and signs of moderate cases, severe cases also showed (I) respiratory distress and respiratory rate (RR) R30 times/minute; (II) oxygen saturation ≤93% under rest state; (III) oxygen partial pressure (PaO2)/oxygen concentration (FiO2) in the arterial blood ≤300 mmHg; and (IV) lung imaging progress >50% in the short term (24–48 h).	The clinical standards for the identification of moderate cases were fever, respiratory track symptoms, and pneumonia imaging (multiple small patches and stromal changes, and the lung extraneous zone was obvious)
30	Wang F et al (94)	All 333 COVID-19 patients were classified as mild, moderate, severe or critically ill category at admission. The diagnostic standard and classifying criteria of COVID-19 were based on the interim guidance from the WHO	All other patients were non-severe
31	Wang K et al (59)	Severe type − adults with any of the following: (1) respiratory distress, RR ⩾30 beats/min, (2) oxygen saturation of ⩽93% during inhalation at rest, (3) an arterial partial pressure of oxygen (PaO2)/fraction of inspired oxygen (FiO2) of ⩽300 mm Hg and (4) chest imaging indicating an obvious ⩾50% progression of infiltrations within 24–48 h.	Light type – mild clinical symptoms with no pneumonia on imaging. Mild/moderate disease type – fever with respiratory symptoms and presence of pneumonia on imaging.
32	Wang W et al(95)	Critical patients and non-critical patients (including severe and mild patients) were categorized based on the 7th edition of the Chinese National Health Commission,6 meeting any of the following criteria:<br>(a)respiratory failure required mechanical ventilation;<br>(b) the patient was prone to shock;<br>(c)the patient with multiple organ failure required ICU treatment.	All other patients were non-severe
33	Wang X et al (41)	For severe pneumonia (meeting any of the following): (1) dyspnoea, respiratory rate of ≥30 breaths/min; (2) peripheral oxygen saturation ≤93% at rest and (3) oxygen partial pressure/oxygen uptake fraction of ≤300 mmHg (1 mmHg = 0.133 kPa).	For non-severe pneumonia: the above criteria were not met.
34	Wu S et al (96)	A total of 270 patients infected with laboratory-identified SARS-CoV-2 were classified into 2 groups, moderately ill and severely or critically ill, according to the Guidance for Corona Virus Disease 2019 (6th edition)	All other patients were non-severe
35	Xia X et al (60)	severe diagnostic criteria: meet the above diagnostic criteria and have any of the following: respiratory distress, respiratory rate (RR) ≥ 30 beats/min; at rest, Refers to pulse oxygen saturation (SPO 2) ≤ 93%; arterial partial pressure of oxygen (PaO 2)/inhaled oxygen concentration (FIO 2) ≤ 300 mmHg	Common diagnostic criteria: fever, respiratory symptoms, and imaging findings of pneumonia
36	Xie G et al (97)	Severe and non-severe cases were defined according to WHO interim guidance.	

37	Xie L et al (98)	Severe cases Adult cases meeting any of the following criteria: (a) respiratory distress (⩾30 breaths/ min); (b) oxygen saturation ⩽93% at rest; (c) arterial partial pressure of oxygen (PaO2)/ fraction of inspired oxygen (FiO2) ⩽300 mmHg (l mmHg = 0.133 kPa).<br>Critical cases Cases meeting any of the following criteria: (a) respiratory failure and requiring mechanical ventilation; (b) shock; (c) with other organ failure that requires ICU care	Mild cases: The clinical symptoms were mild, and there was no sign of pneumonia on imaging.<br>Moderate cases Showing fever and respiratory symptoms with radiological findings of pneumonia.
38	Xue G et al (46)	Mild to moderate or severe-critically ill cases of COVID-19 were diagnosed according to the Guidance for Corona Virus Disease 2019 (7th edition) by the National Health Commission of China	
39	Yang AP et al (61)	Severe patients additionally met at least one of the following conditions: (1) Shortness of breath, RR ≥ 30 times/min, (2) Oxygen saturation (Resting state) ≤ 93%, (3) PaO2/FiO2 ≤ 300 mmHg.	Non-severe patients met all following conditions: (1) Epidemiology history, (2) Fever or other respiratory symptoms, (3) Typical CT image abnormities of viral pneumonia, and (4) Positive result of RT-PCR for SARS-CoV-2 RNA.
40	Zhang Y et al (103)	According to the guidelines for the diagnosis and treatment of novel coronavirus (2019-nCoV) infection by the National Health Commission (Trial Version 5),7 at the time of hospitalisation, patients with one of respiratory rate > 30 breaths/min, SpO2 < 93% on room air, or PaO2/FiO2 ≤ 300 mm Hg, were classified as severe cases and the others were classified as mild cases.	
41	Zhou Y et al (104)	Severe: with any of the following: respiratory distress with RR>30 time/min, oxygen saturation at rest <93%, or PaO2/FiO2<300 mmHg (I mmHg = 0.133 kPa); (4) critically severe: with any of the following: respiratory failure needing mechanical ventilation, shock, or combination with other organ failure needing ICU intensive care.	Moderate: with fever, respiratory symptoms, and imaging presentations of pneumonia;
42	Zhu Z et al (105)	Severe patients should meet at least one of the following criterions: First, shortness of breath with respiration rate (RR) 30 times/min. Second, oxygen saturation 93% in resting state. Third, partial pressure of arterial oxygen (PaO2)-to-fraction of inspired oxygen (FiO2) ratio 300 mm Hg. Obvious lesion progression >50% within 24-48 hours on pulmonary imaging were also recognized as severe cases. Critical cases were defined when one of the following conditions met: First, respiratory failure and require mechanical ventilation. Second, shock occurred. Third, combined with other organ failure and treated in intensive care unit. Mild and moderate cases were defined as non-severe group, while severe and critical patients were categorized as severe group in this study.	All other patients were non-severe

Table S4. The characteristics of included studies comparing severe and non-severe COVID-19 patients.

Table S3

Author	Country	Groups	Sample (N)	M/F (male %)	p	Age (years)	p	DM	p	HT	p	CVD	p	NLR Value	p
						Mean ± SD / Median (IQR)		N (%)		N (%)		N (%)		Mean ± SD	
Asgar M et al	Pakistan	Severe	33	69/31(69)	0.572	52.58±15.68	<0.001	41(41)	0.452	32(32)	0.97	13(13)	0.334	9.11 ± 2.26	<0.001
		Non-severe	67				0.211							3 ± 1.2	
Bastug A et al	Turkey	Non-Severe	145	81/64(55.9)	<0.001	43(18-83)	<0.001	12(8.3)	<0.001	31(21.4)	<0.001	8(3.5)	<0.001	2.4 ± 3.17	<0.001
		Severe	46	26/20(56.5)		71(28-92)		15(32.6)		28(60.9)		12(26.1)		9 ± 8.12	
Chen R et al	China	Critical	48	38/10 (79)	NR	61 ± 14	NR	5 (10)	NR	23 (48)	NR	7 (15)	NR	16.06 ± 3.77	<0.001*
		Severe	155	93/62 (60)		61 ± 14		23 (15)		52 (34)		14 (9)		8.96 ± 2.07	
		Mild/moderate	345	182/163 (53)		67 ± 12		33 (10)		73 (21)		14 (4)		3.37 ± 0.76	
Cheng B et al	China	Non-Severe	205	71/134(34.6)	<0.001	48.95±18.17	<0.001	20(9.7)	<0.003	48(23.4)	<0.002	15(23.4)	0.013	2 ± 0.30	<0.001
		Severe	251	140/111(55.7)		59.89±17.48		50(19.9)		102(40.6)		37(14.7)		3.37 ± 0.6	
Ding X et al	China	Severe	15	9/6 (60)	0.216	67 (55-76)	<0.001*	5 (7)	NR	9 (13)	NR	6 (8)	NR	4.8 ± 2.10	0.002*
		Non-severe	57	24/33 (42)		46 (35-60)								1.9 ± 0.4	
Fu J et al	China	Severe	16	10/6 (63)	0.818	52 ± 13	0.095	4 (5)	NR	7 (9)	NR	NR	NR	6.29 ± 3.72	0.001*
		Mild/moderate	59	35/24 (59)		45 ± 14								2.33 ± 1.22	
Gong J et al	China	Severe	28	16/12 (57)	0.3	64 (55-72)	<0.01*	NR	NR	NR	NR	NR	NR	3.7 ± 1.37	<0.01*
		Non-severe	161	72/89 (45)		45 (33-62)		NR		NR		NR		1.9 ± 0.25	
Guner R et al	Turkey	SARI/Critical	50	33/17 (66)	0.285	62 ± 12	<0.001*	10 (20)	0.127	16 (32)	0.104	20 (40)	0.006*	5.6 ± 9.12	<0.001*
		Mild/pneumonia	172	99/73 (58)		48 ± 16		20 (12)		36 (21)		36 (21)		2.5 ± 4.6	
Hammad R et al	Egypt	Severe	34	28/36(43.7%)	<0.001	60(52-67)	<0.001	NR(26.6)	NR	NR(34.4)	NR	NR	NR	10 ± 2.57	<0.001
		Non-Severe	30			27(24-29.5)							NR	1.23 ± 0.2	
Hu H et al	China	Mild	19	11/8(57.9)	0.796	43(36-64)	0.08	2(10.5)	0.66	3(15.8)	0.032	1(5.3)	1	3.14 ± 0.8	<0.001
		Severe	21	13/8(61.9)		63(48.5-70)		4(19)		10(47.6)		1(4.8)		10.58 ± 4.16	
Kazancioglu S et al	Turkey	Non-Severe	85	52/33(61.2)	0.682	39(16-75)	<0.001	6(7.1)	0.106	12(14.1)	0.003	2(2.4)	0.022	2.31 ± 1.52	<0.001

		Severe	35	20/15(57.1)		59(37-83)		6(17.1)		14(40)		5(14.3)		4.04 ± 3.50	
Kong M et al	China	Severe	87	45/42 (52)	0.592	68 ± 12	0.005*	18 (21)	0.004*	47 (54)	<0.001*	11 (13)	0.195	6.6 ± 1.5	<0.001*
		Mild	123	59/64 (48)		53 ± 16		9 (7)		32 (26)		9 (7)		3.3 ± 0.4	
Liao D et al	China	Critical	86	61/25 (71)	0.0011*	68 (61-78)	<0.0001*	17 (20)	0.017*	28 (33)	0.21	8 (9)	0.085	16.02 ± 3.05	<0.0001*
		Severe	145	76/69 (52)		67 (58-76)		30 (21)		49 (34)		8 (6)		4.71 ± 0.86	
		Moderate	149	69/80 (46)		56 (42-68)		14 (9)		37 (25)		4 (3)		2.67 ± 0.39	
Lin S et al	China	Mild	22	44511	0.448	44±11.3	<0.001	1(4.5)	0.821	3(15.8)	0.172	0	NR	2.55 ± 0.65	<0.001
		Severe	46	29/17		56.4±13.4		3(6.5)		13(32.6)		3(6.5)		9 ± 2.2	
Liu F et al	China	Severe	19	15/4 (79)	0.006*	63 (40-66)	0.086	3 (16)	0.152	6 (32)	0.217	1 (5)	0.54	3.85 ± 0.75	0.010*
		Non-severe	115	48/67 (42)		50 (36-64)		7 (6)		21 (18)		4 (4)		2.7 ± 0.31	
Liu J et al	China	Severe	79	46/33 (58)	0.849	65 (54-71)	0.008*	13 (17)	0.082	37 (47)	0.086	2 (3)	0.54	8.83 ± 1.88	<0.001*
		Common	43	26/17 (61)		55 (38-66)		2 (5)		13 (30)		0 (0)		3.11 ± 0.76	
Liu YP et al	China	Severe	23	47/37(56)	NR	67(31-91)	0.125	3(13)	0.378	10(43.5)	0.001	6(26.1)	0.004	4.3 ± 2.66	<0.001
		Non-severe	61			51(20-83)		4(6.6)		6(9.8)		2(3.3)		5.17 ± 16.52	
Ok F et al	Turkey	Severe	54	24/30 (44)	0.976	68 ± 15	<0.001*	3 (13)	0.378	10 (44)	0.001*	6 (26)	0.004*	6.1 ± 3.78	<0.001*
		Non-severe	85	38/47 (45)		47 ± 16		4 (7)		6 (10)		2 (3)		2.46 ± 1.7	
Qin C et al	China	Severe	286	155/131 (54)	0.242	61 (51-69)	<0.001*	53 (19)	0.152	105 (37)	<0.001*	24 (8)	0.004*	5.5 ± 1.11	<0.001*
		Non-severe	166	80/86 (48)		53 (41-62)		22 (13)		30 (18)		3 (2)		3.2 ± 0.51)	
Sayah W et al	Algeria	Non-severe	73	46/27(63)	0.294	57±13.4	0.0003	4(11)	0.0006	8(22)	0.072	0(0)	NR	3.5 ± 0.58	<0.0001
		Severe	60	57/23(11)		65±13.5		26(45)		29(45)		7(12)		8.2 ± 2.05	
Sayed A et al	Saudi Arabia	Non-severe	660	295/365(44.7)	0.12	35(34.6-37.26)	0.0062	NR	NR	NR	NR	NR	NR	2.85 ± 0.16	0.0014
		Severe	41	22/19(53.66)		45(36.5-52.4)			NR	NR	NR	NR	NR	5.5 ± 1.44	
Seo J et al	South Korea	without ARDS	129	56/73(43)	0.095	56±17	<0.001	19 (15)	0.006	38 (30)	0.002	10 (8)	0.459	NR	-
		with ARDS	37	22/15 (60)		72±11		13 (35)		21 (57)		1 (3)		NR	-
Sepulchre E at al	Belgium	favourable evolution	138	72/66(52.17)	0.0106	60 (47– 74)	0.0136	36(26.28)	0.2286	52(37.68)	0.1058	25(18.12)	0.1724	4 (3-5)	<0.0001
		unfavourable evolution	60	43/17 (71.67)		66.5 (57– 76)		11(18.33)		30(50)		16(26.67)		7 (4-11)	
Shang W et al	China	Severe	139	82/57 (59)	0.008*	64 (54-73)	<0.001*	20 (14)	0.946	45 (32)	0.382	25 (18)	<0.001*	4.75 ± 1.15	<0.001*

		Non-severe	304	138/166 (45)		58 (47-67)		43 (14)		86 (28)		19 (6)		2.38 ± 0.35	
Shi S et al	China	Non-severe	51	27/24(61.1)	NR	58(22-82)	NR	6(11.8)	NR	NR	NR	NR	NR	2.2 ± 0.32	<0.001
		Severe	36	22/14(38.9)		66(39-88)		12(33.3)						6.78 ± 2.01	
Sun S et al	China	Severe	27	18/9 (67)	0.076	62 (53-71)	<0.001*	NR	NR	NR	NR	NR	NR	8.71 ± 2.65	<0.001*
		Common	89	42/47 (47)		47 (37-55)		NR		NR		NR		2.41 ± 0.29	
Wang C et al	China	Moderate	35	17/18(48.6)	0.78	38(16-62)	0.121	2(5.7)	0.441	1(2.9)	0.042	NR	NR	7.93±8.36	<0.001
		Severe	10	6/4(60)		43(28-62)		2(20)		3(30)		NR		29.9±18.7	
Wang F et al	China	Severe	70	45/25 (64)	0.002*	60 (49-64)	<0.001*	NR	NR	NR	NR	NR	NR	2.72 ± 0.62	<0.001*
		Non-severe	253	109/144 (43)		41 (32-56)		NR		NR		NR		1.72 ± 0.22	
Wang K et al	China	Mild-Moderate	33	22/16(57.9)	0.651	45±2	<0.0199	1(2.6)	0.249	6(15.8)	0.279	1(2.6)	0.249	2.14±0.1	0.001
		Severe	5	3/2 (7.9%)		60±6		1(2.6)		2(5.3)		1(2.6)		21.39±20.08	
Wang W et al	China	Severe	50	28/22(56)	0.203	79.5(68-87)	<0.001	11(22)	0.991	31(62)	0.002	14(28)	0.007	9.3 ± 2.32	<0.001
		Non-Severe	73	32/41(43.8)		61(50-68)		16(50-68)		25(34.2)		7(9.5)		1.83 ± 0.12	
Wang X et al	China	Severe	20	49/70(41.2)	0.251	63.5(56-69)	<0.001	25(21)	0.719	47(39.5)	1	12(10.1)	0.617	7.12 ±1.91	<0.001
		Non-severe	111	7/5(58.3)		80(70-85)		3(25)		5(41.7)		2(16.7)		2.21 ± 1.39	
Wu S et al	China	Severe or critical	67	45/22 (67)	NR	66 (54-73)	NR	8 (12)	NR	22 (33)	NR	6 (9)	NR	5.8 ± 2.42	NR
		Moderate	203	86/117 (42)		61 (50-68)		27 (13)		59 (29)		5 (3)		2.2 ± 0.31	
Xia X et al	China	Moderate	32	15/17	NR	62.25±15.07	NR	3(9.35)	NR	9 (28.12)		0 (0)	NR	2.89 (1.77 – 5.56)	0
		severe	31	18/13	NR	64.55±14.88	NR	9(29.03)	NR	15(48.38)		2(6.45)	NR	8.78 (5.76 – 25.10)	
Xie G et al	China	Severe	12	10/2 (83)	0.003*	52 (35-66)	0.092	2 (17)	0.068	4 (33)	0.042*	2 (17)	0.032*	3.52 ± 1.44	0.026*
		Non-severe	85	43/42 (51)		45 (32-60)		3 (4)		16 (19)		5 (6)		2.74 ± 0.32	
Xie L et al	China	Severe	51	29/22 (57)	0.039*	NR	NR	8 (16)	0.0466*	12 (24)	0.379	6 (12)	0.0130*	7.90 ± 10.20	<0.0001*
		Non-severe	322	168/154 (52)		NR		21 (7)		59 (18)		12 (4)		2.93 ± 1.80	
Xue G et al	China	Severe	58	34/24	0.587	64.00(49.75–73.00)	0.527	5(8.62)	0.09	15(25.86)	0.164	6(10.34)	0.164	6.57 ± 1.66	<0.001
		Non-Severe	56	30/26		60.50(52.25–68.75)		11(19.64)		22(39.29)		11(19.64)		3.03 ± 0.63	
Yang AP et al	China	Severe	24	18/6 (75)	0.135	58 ± 12	<0.05*	13 (54)	<0.01*	16 (67)	<0.01*	9 (38)	<0.01*	20.7 ± 24.1	<0.01*
		Non-severe	69	38/31 (55)		42 ± 19		8 (12)		7 (10)		4 (6)		4.8 ± 3.5	

Zhang Y et al	China	Severe	31	20/11 (65)	0.004*	65 ± 13	<0.001*	NR	NR	NR	NR	NR	NR	7.58 ± 7.04	<0.001*
		Mild	84	29/55 (35)		44 ± 15		NR		NR		NR		2.28 ± 1.29	
Zhou Y et al	China	Moderate	140	55/85 (39)	NR	56 ± 14	NR	NR	NR	NR	NR	NR	NR	3.1 ± 2.41	NR
		Severe	123	58/65 (47)		64 ± 14		NR		NR		NR		11.66 ± 27.66	
		Critically severe	41	25/16 (61)		65 ± 13		NR		NR		NR			
Zhu Z et al	China	Severe	16	9/7 (56)	0.457	58 ± 12	0.03*	0 (0)	0.451	8 (50)	0.025*	2 (13)	0.348	5.58 ± 1.96	0.015*
		Non-severe	111	73/38 (66)		50 ± 16		10 (9)		23 (21)		4 (4)		2.75 ± 0.34	
Fei M et al	China	non-critical	52	20/32 (38.46)	0.099	55.7±11.9	0.054	NR	NR	NR	NR	NR	NR	2.66±1.93	0.004
		severe	20	12/8 (60)		64.0±16.8		NR	NR	NR	NR	NR	NR	6.14±4.75	

Table S5. The characteristics of included studies comparing survivors and non-survivors of COVID-19.

Table S5

**Author**	**Coun-try**	**Groups**	**Sample (N)**	**M/F (male%)**	**p**	**Age (years) Mean ± SD / Median (IQR)**	**p**	**DM N (%)**	**p**	**HT N (%)**	**p**	**CVD N (%)**	**p**	**NLR Mean ± SD**	**p**
Abrishami A et al	Iran	survivor	83	53/30(63.8)	0.04*	55.2±15.6		20(24.1)		31(37.3)		16(19.3)		3.02 ± 0.34	
		non survivor	17	15/2(88.2)		57±13.2	0.637	1(5.8)	0.71	2(11.7)	0.04	5(29.5)	0.34	5.81 ± 1.9	0.02
Acar et al	Turkey	Survivor	129	42/87	<0.05*	57.91		20		43		9		9.4 ± 13.85	
		non survivor	19	014/5		69.89	<0.05*	6	>0.05	11	<0.05*	2	>0.05	27.15 ± 14.71	<0.05*
Asghar et al	Pakistan	survivor	NR	NR	NR	NR	NR	NR	NR	NR	NR	NR	NR	5.45 ± 5.40	NR
		non survivor	NR	NR	NR	NR	NR	NR	NR	NR	NR	NR	NR	12.65 ± 10.37	NR
BG et al	India	survivor	75	44/31	0.56	43±13.6	<0.001*	17	not re-ported	19	not re-ported	5	not re-ported	8.88 ±3.84	
		non survivor	25	013/12		59.1±11.5		19		5		5		4.87 ±3.7	0.004
Chen F et al	China	survivor	577	297/ 280	0.038	63	<0.001*	96		227		55		3.38 ± 0.69	
		non survivor	104	65/ 39		82		18	0.866	66	<0.001*	25	<0.001*	12.27 ± 2.79	<0.001*
Chen L et al	China	Survivor	1651	781/870 (47)	<0.001*	57 (43-66)	<0.001*	203 (12)		475 (29)		205 (12)[$]		3 ± 0.33	
		Non-survivor	208	153/55 (74)		70 (63-78)		59 (28)	<0.001*	104 (50)	<0.001*	62 (30)[$]	<0.001*	11 ± 2.33	<0.001*
Chen R et al	China	Survivor	445	244/201 (55)	<0.05*	54 ± 14		41 (9)		103 (23)		24 (5)		3.71 ± 0.87	
		Non-survivor	103	69/34 (67)		67 ± 12	<0.01*	20 (19)	<0.01*	45 (44)	<0.01*	11 (11)	>0.05	13.45 ± 2.37	<0.001*
Cheng B et al	China	Survivor	67	22/45 (33)	0.002*	71 ± 7		11 (16)		39 (58)		11 (16)		4.1 ± 2.9	
		Non-survivor	51	31/20 (61)		73 ± 7	0.048*	16 (31)	0.055	25 (49)	0.321	12 (24)	0.334	13.3 ± 14.9	<0.001*

Goya R L et al	Spain	survivor	465	317/ 148	0.54	52(44-58)		49(10.5)		117(25.2)		not re-ported		4.2 ± 0.93	
		non survivor	36	025/11		65(57-72)	<0.001 *	7(19.4)	0.11	22(61.1)	<0.001*	not re-ported	not re-ported	16.9 ± 5.25	<0.001*
Güneysu F et al	Turkey	Survivor	84	50/34(59.5)	0.388	72.02 ±11.39		NR	NR	NR	NR	NR	NR	6.43 ± 1.80	
		Non-survivor	85	45/40(52.9)		56.75 ±19.38	<0.001	NR	NR	NR	NR	NR	NR	2.88 ± 0.54	<0.001
Huang J et al	China	Survivor	283	149/134 (53)	0.318	53 ± 17	<0.001 *	31 (11)		63 (22)		14 (5)		3.3 ± 4.3	
		Non-survivor	16	11/5 (69)		69 ± 10		4 (25)	0.103	11 (69)	<0.001*	4 (25)	0.011*	13.3 ± 14.3	0.013*
Li L et al	China	Survivor	68	26/42 (38)	0.098	44 ± 13		6 (9)		0 (0)		0 (0)		2.3 ± 0.55	<0.0001 *
		Non-survivor	25	15/10 (60)		69 ± 11	<0.01*	5 (20)	0.158	5 (20)	NR	4 (16)	NR	4.25 ± 0.95	
Luo X et al	China	Survivor	214	99/115 (46)	0.025*	51 (37-63)		27 (13)		37 (17)		13 (6)		2.96 ± 0.41	
		Non-survivor	84	51/33 (61)		71 (64-80)		18 (21)		49 (58)	<0.001*	13 (16)	0.010*	8.17 ± 0.79	<0.001*
		non survivor	43	31/12 (72.0)		63.81	<0.001 *	21	0.056	NR					
Sanchez A et al	Mexico	Survivor	111	74/37(67)	0.162	51		37(33.3)		45(45.1)		24(22.1)		8.31 ± 1.47	
		Non-survivor	83	63/20 (76)		62	0.019	37(44.6)	0.111	49(59.4)	0.072	16(19.1)	0.706	17.66 ± 2.71	0.001
Sepulchre E et al	Belgium	Survivor	138		0.01	60(47-74)		26.28		37.68		18.12		4 ± 0.33	
		Non-survivor	60	115/83(58)		66.5(57-76)	0.013	18.33	0.22	50	0.105	26.67	0.17	7 ± 1.75	<0.0001
Ullah	USA	NLR < 10	141	66 (80.50%)	0.04	63.6		49 (35)		93 (66)		27 (19)		NR	
		NLR > 11	26	16 (19.50%)		61.6	0.65	10 (39)	0.716	14 (54)	0.16	2 (8)	0.65	NR	NR
Tatum	USA	NLR ≤ 4.94	62		0.454	56.1 ± 15.2		NR	NR	NR	NR	NR	NR	NR	
		NLR > 4.94	57	57/68 (21.6)		62.1 ± 14.1	0.060	NR	NR	NR	NR	NR	NR	NR	NR
Liu Y	China	NLR Tertile 1 (0.54–2.21)	82	24 (29)	NR	48 ± 16	<0.001	5 (6)	0.131	11 (13)	0.005	5 (6)	0.100	NR	NR

		NLR Tertile 2 (2.21–4.82)	81	40 (49)		53 ± 17		6 (7)		14 (17)		3 (4)		NR	
		NLR Tertile 3 (4.85–88.09)	82	50 (61)		61 ± 15		12 (15)		27 (33)		10 (12)		NR	
Wang X et al	China	survivor	119	49/ 70		63.5		25		47		12		1.95 ± 0.19	
		non survivor	12	7/-5	0.251	80	<0.001	3		5		2		15.01 ± 5.01	
		Moderate	26	15/11		57.96 ± 14.51	*	3	0.719	3	1	3	0.617		<0.001*
Yan X et al	China	Survivor	964	466/498 (48)	0.018*	62 (50-70)		97 (11)		215 (22)		65 (7)		4.11 ± 0.94	
		Non-survivor	40	27/13 (68)		68 (58-79)	0.007*	10 (25)	0.003*	20 (50)	<0.001*	10 (25)	<0.001*	49.06 ± 10.99	<0.001*
Yang Q et al	China	Survivor	176	82/94 (47)	0.077	50 ± 15	<0.001	28 (16)		47 (27)		6 (3)		2.98 ± 0.63	
		Non-survivor	50	31/19 (62)		68 ± 16	*	17 (34)	0.008*	37 (74)	<0.001*	7 (14)	0.010*	6.18 ± 2.3	<0.001*
Yavuz B et al	Turkey	survivor	91	56/35(61.5)	0.166	70.1 ±7.9		38(41.8)		52(57.1)		14(15.4)		4.5 ±4.6	
		non survivor	22	17/5(77.3)		73.1 ±7.6	0.084	11(50)	0.48	11(50)	0.545	3(13.6)	0.837	9.9 ±10.5	0.002
Ye W et al	China	Survivor	297	137/160 (46)	0.002*	60 (50-67)	<0.001	41 (14)		73 (25)		5 (2)		2.88 ± 0.82	
		Non-survivor	52	36/16 (69)		69 (63-76)	*	16 (31)	0.004*	30 (58)	<0.001*	11 (21)	<0.001*	14.96 ± 4.51	<0.001*
Zhang N et al	China	Survivor	50	36/14 (72)	NR	63 ± 11		5 (10)		18 (36)		11 (22)		8.4 ± 7.5	
		Non-survivor	10	7/3 (70)		71 ± 9	0.044*	4 (40)	0.025*	4 (40)	0.811	3 (30)	0.587	18.7 ± 16.6	0.030*
Zhang S et al	China	Survivor	420	213/207 (51)	NR	59 (48-67)		60 (14)		107 (26)		53 (13)		3.91 ± 0.78	
		Non-survivor	96	72/24 (75)		67 (61-74)	NR	16 (17)	NR	31 (32)	NR	14 (15)	NR	10.99 ± 2.21	<0.001*

Table S6. Risk of bias assessment of all included studies using the Newcastle-Ottawa Scale (NOS).

Table S5						
Author	Year of Publication	Study Location	Selection	Comparability	Outcome/Exposure	NOS SCORE
Abrishami A et al	2021	Iran	***	*	***	7
Acar et al	2021	Turkey	***	*	***	7
Asgar M et al	2020	Pakistan	***	*	***	7
Bastug A et al	2020	Turkey	***	*	***	7
BG et al	2021	India	***	*	***	7
Chen F et al	2020	China	***	*	***	7
Chen L et al	2020	China	****	**	***	9
Chen R et al	2020	China	****	**	***	9
Cheng B et al	2020	China	***	**	***	8
Ding X et al	2020	China	**	**	***	8
Fei M et al	2020	China	**	*	***	5
Fu J et al	2020	China	**	*	***	6
Gong J et al	2020	China	***	*	***	7
Goya R L et al	2020	Spain	**	*	***	6
Guner R et al	2020	Turkey	**	*	***	6
Güneysu F et al	2020	Turkey	***	*	***	7

Hammad R et al	2021	Egypt	***	*	***	7
Hu H et al	2020	China	**	*	***	6
Huang J et al	2020	China	***	**	***	8
Kazancioglu S et al	2020	Turkey	***	**	***	8
Kong M et al	2020	China	**	**	***	7
Li L et al	2020	China	***	*	***	7
Liao D et al	2020	China	**	**	***	7
Lin S et al	2020	China	***	*	***	7
Liu F et al	2020	China	***	**	***	8
Liu J et al	2020	China	**	**	***	7
Liu YP et al	2020	China	***	**	***	8
Liu Y et al	2020	China	***	*	***	7
Luo X et al	2020	China	***	**	***	8
Ma Y et al	2020	China	**	**	**	6
Ok F et al	2020	Turkey	**	**	***	7
Qin C et al	2020	China	**	-	***	5
Ramesh J et al	2021	India	***	**	***	8
Sanchez A et al	2020	Mexico	***	*	***	6
Sayah W et al	2021	Algeria	***	**	***	8
Sayed A et al	2021	Saudi Arabia	***	*	***	7

Seo J et al	2021	Korea	***	*	***	7
Sepulchre E at al	2020	Belgium	***	*	***	7
Shang W et al	2020	China	**	**	***	7
Shi S et al	2021	China	***	*	**	6
Sun S et al	2020	China	**	-	***	5
Ullah W et al	2020	USA	**	*	***	6
Wang C et al	2020	China	***	*	***	7
Wang F et al	2020	China	***	**	***	8
Wang K et al	2021	China	***	*	***	7
Wang W et al	2021	China	***	*	***	7
Wang X et al	2020	China	***	*	***	7
Wu S et al	2020	China	**	**	***	7
Xia X et al	2020	China	**	**	***	8
Xie G et al	2020	China	**	-	***	5
Xie L et al	2020	China	**	-	***	5
Xu J et	2020	China	**	-	***	5
Xue G et al	2020	China	***	*	***	7
Yan X et al	2020	China	***	**	***	8
Yang AP et al	2020	China	**	**	***	7
Yang Q et al	2020	China	***	**	***	8

Yavuz B et al	2021	Turkey	***	**	***	9
Ye W et al	2020	China	***	**	***	8
Zhang N et al	2020	China	***	-	***	6
Zhang S et al	2020	China	***	*	***	7
Zhang Y et al	2020	China	**	**	***	7
Zhou Y et al	2020	China	**	-	***	5
Zhu Z et al	2020	China	**	**	***	7

Figure S1A: Forest plot of studies comparing severe disease with non-severe disease stratified by severity criteria

Study or Subgroup	Severe Mean	Severe SD	Severe Total	Non-Severe Mean	Non-Severe SD	Non-Severe Total	Weight	Std. Mean Difference IV, Random, 95% CI
**1.1.1 Based on need for IMV**								
Fu J et al (81)	6.29	3.72	16	2.33	1.22	59	2.5%	1.95 [1.31, 2.59]
Lin S et al	9	2.2	46	2.55	0.65	22	2.5%	3.44 [2.66, 4.22]
Liu F et al (91)	3.85	0.75	19	2.7	0.31	115	2.6%	2.86 [2.26, 3.46]
Liu YP et al	16.52	5.17	61	4.3	2.66	23	2.5%	2.61 [1.99, 3.24]
Shang W et al (55)	4.76	1.15	139	2.38	0.35	304	2.7%	3.37 [3.07, 3.67]
Shi S et al (56)	6.78	2.01	36	2.2	0.32	51	2.5%	3.46 [2.78, 4.13]
Wang W et al (95)	9.3	2.32	50	1.83	0.12	73	2.5%	5.02 [4.29, 5.75]
Xie L et al (98)	7.9	10.2	51	2.94	1.8	322	2.7%	1.21 [0.90, 1.51]
Zhu Z et al (105)	5.58	1.96	16	2.75	0.35	111	2.5%	3.73 [3.03, 4.43]
Subtotal (95% CI)			434			1080	23.0%	3.05 [2.25, 3.86]
Heterogeneity: Tau² = 1.41; Chi² = 169.96, df = 8 (P < 0.00001); I² = 95%								
Test for overall effect: Z = 7.46 (P < 0.00001)								
**1.1.2 Based on oxygenation needs (SaO2/PaO2/FiO2)**								
Cheng B et al	3.37	0.6	251	2	0.3	205	2.7%	2.80 [2.54, 3.06]
Guner R et al	9.12	5.6	50	2.5	4.6	172	2.7%	1.36 [1.02, 1.70]
Hammad R et al	10	2.57	34	1.23	0.2	30	2.4%	4.61 [3.65, 5.57]
Kazancioglu S et al	4.04	3.5	35	2.31	1.52	85	2.6%	0.76 [0.35, 1.16]
Kong M et al	6.6	1.5	87	3.3	0.4	123	2.6%	3.25 [2.83, 3.67]
Liao D et al	16.02	3.05	86	2.67	0.39	149	2.5%	7.12 [6.42, 7.82]
Liu J et al	8.83	1.88	79	3.11	0.76	43	2.6%	3.60 [3.01, 4.19]
Ok F et al	6.1	3.78	54	2.46	1.7	85	2.6%	1.34 [0.96, 1.72]
Qin C et al	5.5	1.11	286	3.2	0.51	166	2.7%	2.45 [2.20, 2.70]
Sayah W et al	8.2	2.05	60	3.5	0.58	73	2.6%	3.24 [2.72, 3.76]
Sun S et al	8.71	2.65	27	2.41	0.28	89	2.5%	4.85 [4.09, 5.62]
Wang C et al	29.9	18.7	10	7.93	8.36	35	2.5%	1.90 [1.09, 2.72]
Wang F et al	2.72	0.62	70	1.72	0.22	253	2.7%	2.87 [2.53, 3.22]
Wang K et al	21.39	20.08	5	2.14	0.1	33	2.2%	2.82 [1.66, 3.97]
Wang X et al	7.12	1.91	20	2.21	1.39	111	2.6%	3.30 [2.68, 3.93]
Wu S et al	5.8	2.42	67	2.2	0.31	203	2.6%	2.92 [2.55, 3.29]
Xie G et al	3.52	1.44	12	2.74	0.32	85	2.5%	1.35 [0.71, 1.98]
Yang AP et al	20.7	24.1	24	4.8	3.5	69	2.6%	1.26 [0.76, 1.76]
Zhang Y et al	7.58	7.04	31	2.28	1.25	84	2.6%	1.39 [0.94, 1.84]
Zhou Y et al	27.66	11.66	164	3.1	2.41	140	2.7%	2.81 [2.49, 3.13]
Subtotal (95% CI)			1452			2233	51.4%	2.77 [2.27, 3.27]
Heterogeneity: Tau² = 1.20; Chi² = 491.56, df = 19 (P < 0.00001); I² = 96%								
Test for overall effect: Z = 10.93 (P < 0.00001)								
**1.1.3 Based on need for ICU admission only**								
Asghar et al	9.11	2.26	33	3	1.2	67	2.5%	3.73 [3.06, 4.41]
Bastug A et al	9	8.12	46	2.4	3.17	145	2.7%	1.36 [1.00, 1.72]
Sayed A et al (58)	5.5	1.44	41	2.85	0.16	660	2.6%	7.01 [6.52, 7.49]
Sepulchre E et al (54)	7.25	0.24	60	4	0.62	138	2.5%	6.05 [5.38, 6.73]
Subtotal (95% CI)			180			1010	10.3%	4.53 [1.57, 7.50]
Heterogeneity: Tau² = 9.06; Chi² = 388.28, df = 3 (P < 0.00001); I² = 99%								
Test for overall effect: Z = 3.00 (P = 0.003)								
**1.1.4 Based on Hematological parameters only**								
Chen R et al	16.06	3.77	48	3.37	0.76	345		Not estimable
Xue G et al	6.57	1.66	58	3.03	0.63	56	2.6%	2.78 [2.26, 3.30]
Subtotal (95% CI)			58			56	2.6%	2.78 [2.26, 3.30]
Heterogeneity: Not applicable								
Test for overall effect: Z = 10.50 (P < 0.00001)								
**1.1.5 Based on radiological parameters**								
Ding X et al	4.8	2.1	15	1.9	0.4	57	2.5%	2.85 [2.11, 3.60]
Fei M et al	6.14	4.75	20	2.66	1.93	52	2.6%	1.16 [0.61, 1.71]
Subtotal (95% CI)			35			109	5.1%	1.99 [0.32, 3.65]
Heterogeneity: Tau² = 1.33; Chi² = 12.90, df = 1 (P = 0.0003); I² = 92%								
Test for overall effect: Z = 2.34 (P = 0.02)								
**1.1.6 Not Mentioned**								
Gong J et al	3.7	1.37	28	1.9	0.25	161	2.6%	3.15 [2.63, 3.66]
Hu H et al	10.58	4.16	21	3.14	0.8	19	2.4%	2.38 [1.55, 3.21]
Xia X et al	12.1	4.83	31	3.27	1.12	32	2.5%	2.51 [1.84, 3.18]
Subtotal (95% CI)			80			212	7.6%	2.75 [2.25, 3.25]
Heterogeneity: Tau² = 0.08; Chi² = 3.50, df = 2 (P = 0.17); I² = 43%								
Test for overall effect: Z = 10.74 (P < 0.00001)								
**Total (95% CI)**			2239			4700	100.0%	2.97 [2.55, 3.40]
Heterogeneity: Tau² = 1.70; Chi² = 1174.08, df = 38 (P < 0.00001); I² = 97%								
Test for overall effect: Z = 13.87 (P < 0.00001)								
Test for subgroup differences: Chi² = 2.68, df = 5 (P = 0.75), I² = 0%								

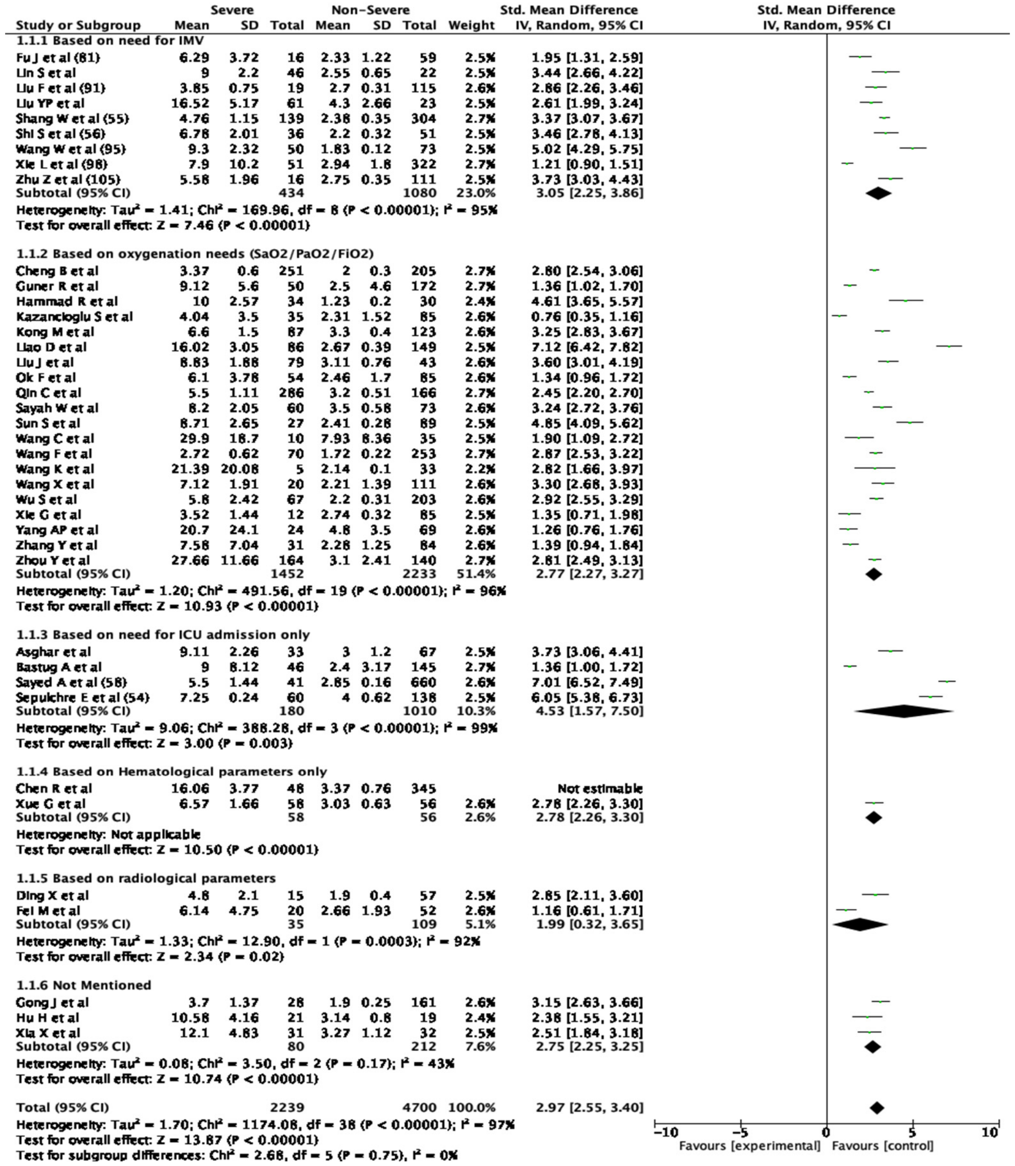

Figure S1B: Forest plot of studies comparing severe disease with non-severe disease stratified by region

Study or Subgroup	Severe Mean	Severe SD	Severe Total	Non-Severe Mean	Non-Severe SD	Non-Severe Total	Weight	Std. Mean Difference IV, Random, 95% CI
**1.1.1 Asia**								
Asghar et al	9.11	2.26	33	3	1.2	67	2.5%	3.73 [3.06, 4.41]
Bastug A et al	9	8.12	46	2.4	3.17	145	2.6%	1.36 [1.00, 1.72]
Cheng B et al	3.37	0.6	251	2	0.3	205	2.6%	2.80 [2.54, 3.06]
Chen R et al	16.06	3.77	48	3.37	0.77	345	2.5%	8.48 [7.81, 9.15]
Ding X et al	4.8	2.1	15	1.9	0.4	57	2.5%	2.85 [2.11, 3.60]
Fei M et al	6.14	4.75	20	2.66	1.93	52	2.5%	1.16 [0.61, 1.71]
Fu J et al	6.29	3.72	16	2.33	1.22	59	2.5%	1.95 [1.31, 2.59]
Gong J et al	3.7	1.37	28	1.9	0.25	161	2.5%	3.15 [2.63, 3.66]
Guner R et al	5.6	9.12	50	2.5	4.6	172	2.6%	0.52 [0.20, 0.84]
Hu H et al	10.58	4.16	21	3.14	0.8	19	2.4%	2.38 [1.55, 3.21]
Kazancioglu S et al	4.04	3.5	35	2.31	1.52	85	2.5%	0.76 [0.35, 1.16]
Kong M et al	6.6	1.5	87	3.3	0.4	123	2.5%	3.25 [2.83, 3.67]
Liao D et al	16.02	3.05	86	2.67	0.3983	149	2.5%	7.12 [6.42, 7.82]
Lin S et al	9	2.2	46	2.55	0.65	22	2.4%	3.44 [2.66, 4.22]
Liu F et al	3.85	0.75	19	2.7	0.31	115	2.5%	2.86 [2.26, 3.46]
Liu J et al	8.83	1.88	79	3.11	0.76	43	2.5%	3.60 [3.01, 4.19]
Liu YP et al	4.3	2.66	23	5.17	16.52	61	2.5%	-0.06 [-0.54, 0.42]
Ok F et al	6.1	3.78	54	2.46	1.7	85	2.5%	1.34 [0.96, 1.72]
Qin C et al	5.5	1.11	286	3.2	0.51	166	2.6%	2.45 [2.20, 2.70]
Sayed A et al	5.5	1.44	41	2.85	0.16	660	2.5%	7.01 [6.52, 7.49]
Shang W et al	4.75	1.15	139	2.38	0.35	304	2.6%	3.35 [3.05, 3.65]
Shi S et al	6.78	2.01	36	2.2	0.32	51	2.5%	3.46 [2.78, 4.13]
Sun S et al	8.71	2.65	27	2.41	0.29	89	2.4%	4.85 [4.08, 5.61]
Wang C et al	29.9	18.7	10	7.93	8.36	35	2.4%	1.90 [1.09, 2.72]
Wang F et al	2.72	0.62	70	1.72	0.22	253	2.6%	2.87 [2.53, 3.22]
Wang K et al	21.39	20.08	5	2.14	0.1	33	2.3%	2.82 [1.66, 3.97]
Wang W et al	9.3	2.32	50	1.83	0.12	73	2.5%	5.02 [4.29, 5.75]
Wang X et al	7.12	1.91	20	2.21	1.39	111	2.5%	3.30 [2.68, 3.93]
Wu S et al	5.8	2.42	67	2.2	0.31	203	2.6%	2.92 [2.55, 3.29]
Xia X et al	12.105	4.835	31	3.2775	1.1223	32	2.5%	2.50 [1.83, 3.17]
Xie G et al	3.52	1.44	12	2.74	0.32	85	2.5%	1.35 [0.71, 1.98]
Xie L et al	7.9	10.2	51	2.93	1.8	322	2.6%	1.21 [0.90, 1.52]
Xue G et al	6.57	1.66	58	3.03	0.63	56	2.5%	2.78 [2.26, 3.30]
Yang AP et al	20.7	24.1	24	4.8	3.5	69	2.5%	1.26 [0.76, 1.76]
Zhang Y et al	7.58	7.04	31	2.28	1.29	84	2.5%	1.39 [0.94, 1.84]
Zhou Y et al	11.66	27.66	164	3.1	2.41	140	2.6%	0.42 [0.19, 0.65]
Zhu Z et al	5.58	1.96	16	2.75	0.34	111	2.5%	3.75 [3.05, 4.45]
**Subtotal (95% CI)**			**2095**			**4842**	**92.6%**	**2.83 [2.30, 3.37]**
Heterogeneity: Tau² = 2.64; Chi² = 1862.97, df = 36 (P < 0.00001); I² = 98%								
Test for overall effect: Z = 10.43 (P < 0.00001)								
**1.1.2 Africa**								
Hammad R et al	10	2.57	34	1.23	0.2	30	2.4%	4.61 [3.65, 5.57]
Sayah W et al	8.2	2.05	60	3.5	0.58	73	2.5%	3.24 [2.72, 3.76]
**Subtotal (95% CI)**			**94**			**103**	**4.9%**	**3.86 [2.53, 5.20]**
Heterogeneity: Tau² = 0.78; Chi² = 6.01, df = 1 (P = 0.01); I² = 83%								
Test for overall effect: Z = 5.68 (P < 0.00001)								
**1.1.3 Europe**								
Sepulchre E et al	7.25	0.24	60	4	0.6124	138	2.5%	6.12 [5.44, 6.80]
**Subtotal (95% CI)**			**60**			**138**	**2.5%**	**6.12 [5.44, 6.80]**
Heterogeneity: Not applicable								
Test for overall effect: Z = 17.64 (P < 0.00001)								
**Total (95% CI)**			**2249**			**5083**	**100.0%**	**2.97 [2.45, 3.49]**
Heterogeneity: Tau² = 2.75; Chi² = 2015.24, df = 39 (P < 0.00001); I² = 98%								
Test for overall effect: Z = 11.14 (P < 0.00001)								
Test for subgroup differences: Chi² = 55.84, df = 2 (P < 0.00001), I² = 96.4%								

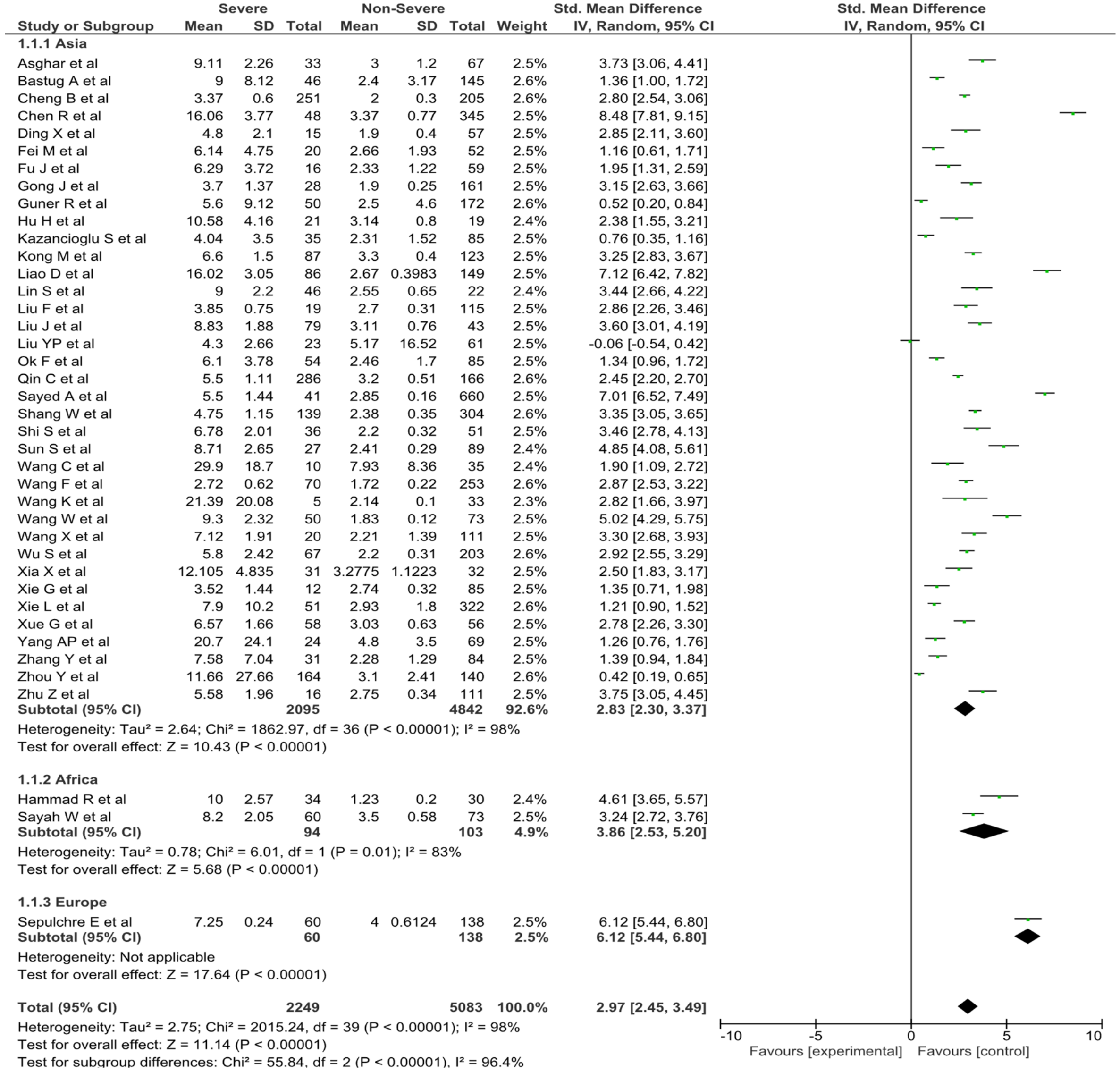

Study or Subgroup	Non-survivors Mean	Non-survivors SD	Non-survivors Total	Survivors Mean	Survivors SD	Survivors Total	Weight	Std. Mean Difference IV, Random, 95% CI
**1.1.1 Asia**								
Abrishami A et al	5.81	1.9	17	3.02	0.34	83	4.5%	3.34 [2.64, 4.05]
Acar et al	27.15	14.71	19	9.4	13.85	129	4.6%	1.27 [0.76, 1.77]
Asghar et al	12.65	10.37	22	5.45	5.4	78	4.6%	1.05 [0.56, 1.55]
BG et al	4.87	3.7	25	8.88	3.84	75	4.6%	−1.05 [−1.52, −0.57]
Chen F et al	12.27	2.79	104	3.38	0.69	577	4.6%	7.05 [6.62, 7.48]
Cheng B et al	13.3	14.9	51	4.1	2.9	67	4.6%	0.91 [0.53, 1.29]
Chen L et al	11	2.33	208	3	0.33	1651	4.6%	9.54 [9.21, 9.88]
Chen R et al	13.45	2.37	103	3.71	0.87	445	4.6%	7.54 [7.04, 8.03]
Huang J et al	13.3	14.3	16	3.3	4.3	283	4.6%	1.89 [1.36, 2.42]
Li L et al	4.25	0.95	25	2.3	0.55	68	4.5%	2.85 [2.23, 3.47]
Luo X et al	8.17	0.79	84	2.96	0.41	214	4.5%	9.55 [8.74, 10.36]
Wang X et al	15.01	5.01	12	1.95	0.19	119	4.5%	8.81 [7.57, 10.04]
Yang Q et al	6.18	2.3	50	2.98	0.63	176	4.6%	2.63 [2.23, 3.03]
Yan X et al	49.06	10.99	40	4.11	0.94	964	4.5%	19.07 [18.17, 19.96]
Yavuz B et al	9.9	10.5	22	4.5	4.6	91	4.6%	0.87 [0.39, 1.35]
Ye W et al	14.94	4.51	52	2.88	0.82	297	4.5%	6.38 [5.82, 6.93]
Zhang N et al	18.7	16.6	10	8.4	7.5	50	4.5%	1.07 [0.36, 1.78]
Zhang S et al	10.99	2.21	96	3.91	0.78	420	4.6%	5.98 [5.55, 6.41]
Subtotal (95% CI)			956			5787	81.8%	4.92 [3.04, 6.80]
Heterogeneity: $Tau^2 = 16.47$; $Chi^2 = 4153.56$, $df = 17$ ($P < 0.00001$); $I^2 = 100\%$								
Test for overall effect: $Z = 5.13$ ($P < 0.00001$)								
**1.1.2 Europe**								
Goya R L et al	16.9	5.25	36	4.2	0.93	465	4.5%	7.66 [7.08, 8.25]
Güneysu F et al	2.88	0.54	85	6.43	1.8	84	4.6%	−2.67 [−3.08, −2.25]
Sepulchre E et al	7	1.75	43	4	0.33	155	4.6%	3.47 [2.99, 3.95]
Subtotal (95% CI)			164			704	13.7%	2.82 [−3.09, 8.73]
Heterogeneity: $Tau^2 = 27.20$; $Chi^2 = 872.93$, $df = 2$ ($P < 0.00001$); $I^2 = 100\%$								
Test for overall effect: $Z = 0.94$ ($P = 0.35$)								
**1.1.3 North America**								
Sanchez A et al	17.66	2.71	83	8.31	1.47	111	4.6%	4.45 [3.92, 4.98]
Subtotal (95% CI)			83			111	4.6%	4.45 [3.92, 4.98]
Heterogeneity: Not applicable								
Test for overall effect: $Z = 16.46$ ($P < 0.00001$)								
Total (95% CI)			1203			6602	100.0%	4.61 [2.89, 6.33]
Heterogeneity: $Tau^2 = 16.82$; $Chi^2 = 5318.92$, $df = 21$ ($P < 0.00001$); $I^2 = 100\%$								
Test for overall effect: $Z = 5.26$ ($P < 0.00001$)								
Test for subgroup differences: $Chi^2 = 0.52$, $df = 2$ ($P = 0.77$), $I^2 = 0\%$								

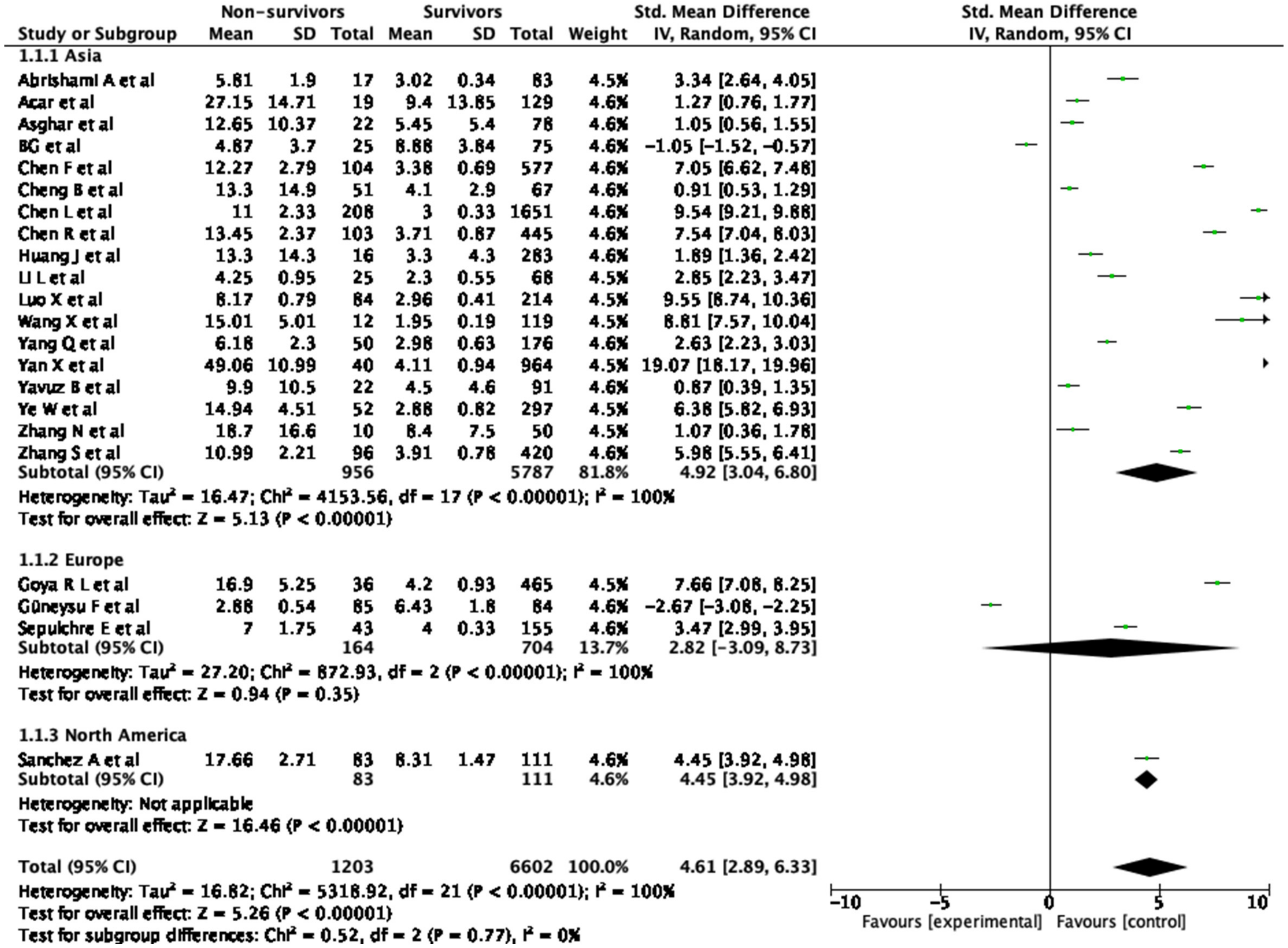

Figure S2: Forest plot of studies comparing non-survivors with survivors, stratified by region

Figure S3 A: Forest plot of the sensitivity and specificity and DOR of NLR for predicting disease severity in patients with COVID-19.

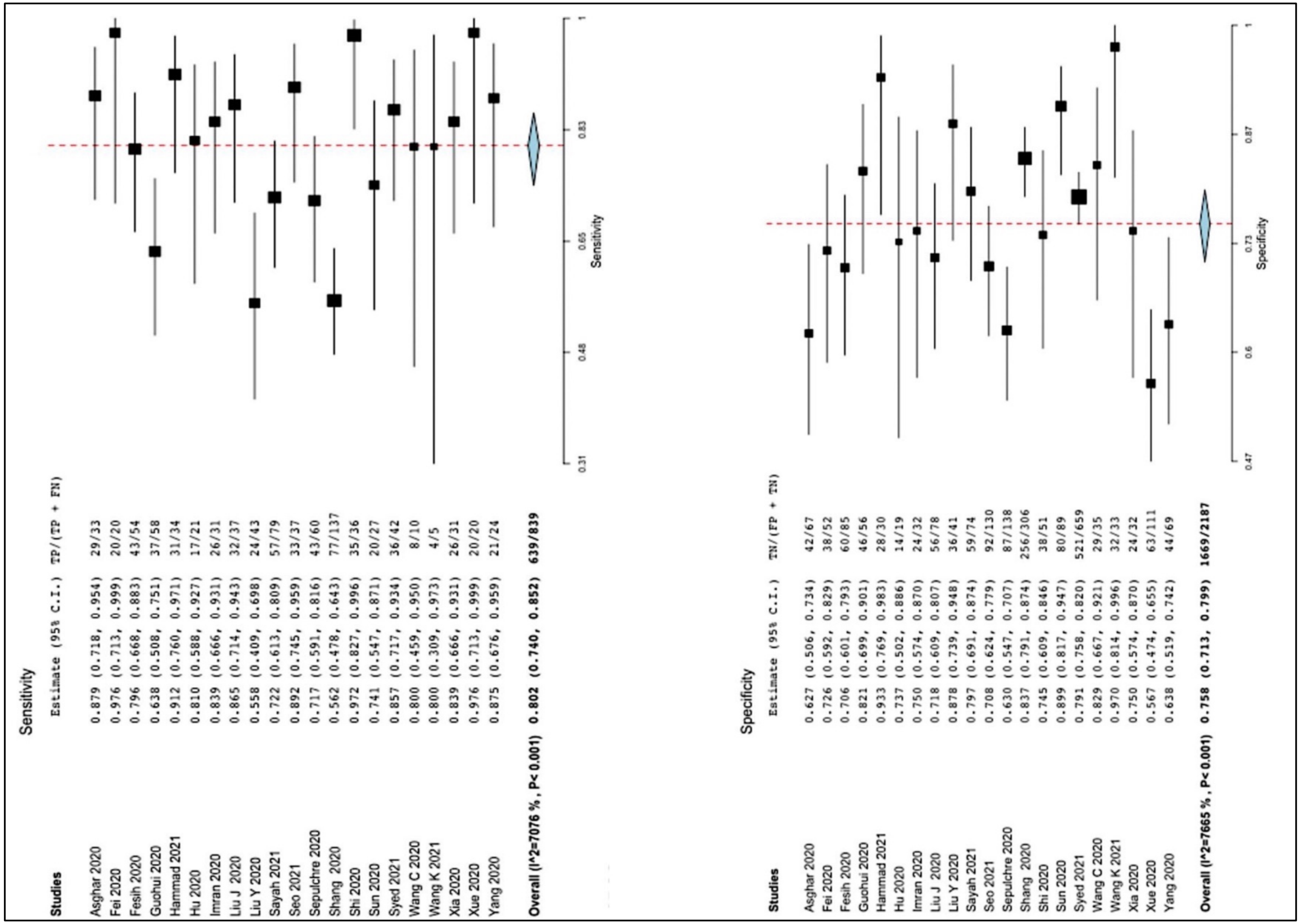

Studies	Estimate (95% C.I.)	(TP * TN)/(FP * FN)
Asghar 2020	12.180 (3.831, 38.725)	1218/100
Fei 2020	108.862 (6.174, 1919.371)	760/0
Fesih 2020	9.382 (4.173, 21.091)	2580/275
Guohui 2020	8.105 (3.400, 19.318)	1702/210
Hammad 2021	144.667 (22.504, 929.982)	868/6
Hu 2020	11.900 (2.674, 52.959)	238/20
Imran 2020	15.600 (4.481, 54.314)	624/40
Liu J 2020	16.291 (5.623, 47.198)	1792/110
Liu Y 2020	9.095 (2.990, 27.661)	864/95
Sayah 2021	10.191 (4.812, 21.584)	3363/330
Seo 2021	19.974 (6.620, 60.267)	3036/152
Sepulchre 2020	4.315 (2.232, 8.343)	3741/867
Shang 2020	6.571 (4.175, 10.342)	19712/3000
Shi 2020	102.308 (12.716, 823.138)	1330/13
Sun 2020	25.397 (8.432, 76.494)	1600/63
Syed 2021	22.652 (9.355, 54.853)	18756/828
Wang C 2020	19.333 (3.256, 114.812)	232/12
Wang K 2021	128.000 (6.631, 2470.861)	128/1
Xia 2020	15.600 (4.481, 54.314)	624/40
Xue 2020	53.680 (3.167, 909.817)	1260/0
Yang 2020	12.320 (3.339, 45.456)	924/75
**Overall (I^2=4565 % , P=0.007)**	**13.639 (9.777, 19.028)**	**65352/6237**

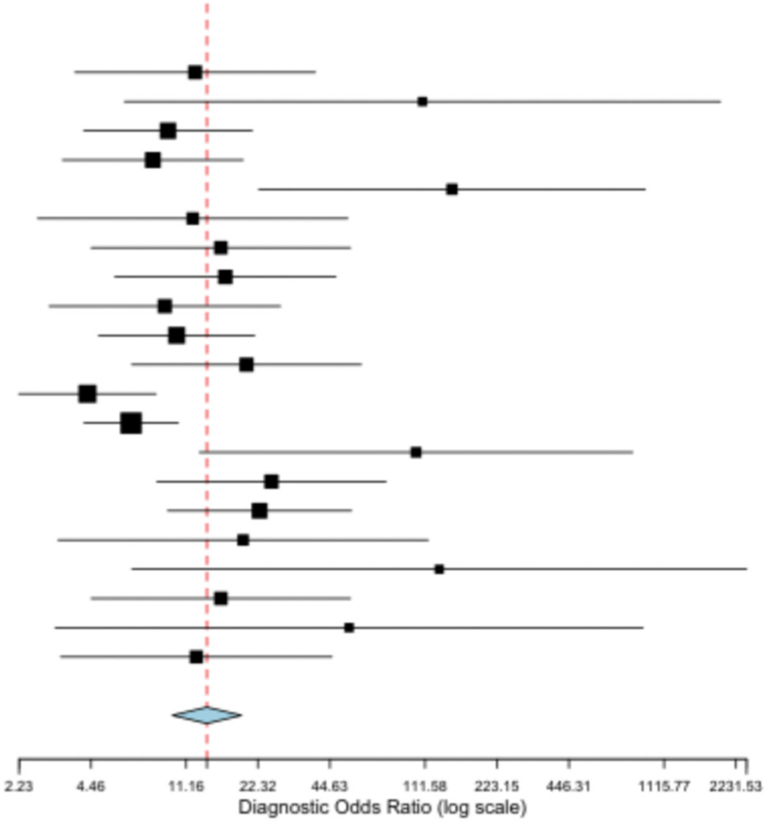

Forest plot of the odds of NLR for predicting disease severity in patients with COVID-19. The pooled OR was 13.639 (95% CI: 9.777 – 19.028).

Figure S3 B: Forest plot of the sensitivity and specificity and DOR of NLR for predicting disease mortality in patients with COVID-19.

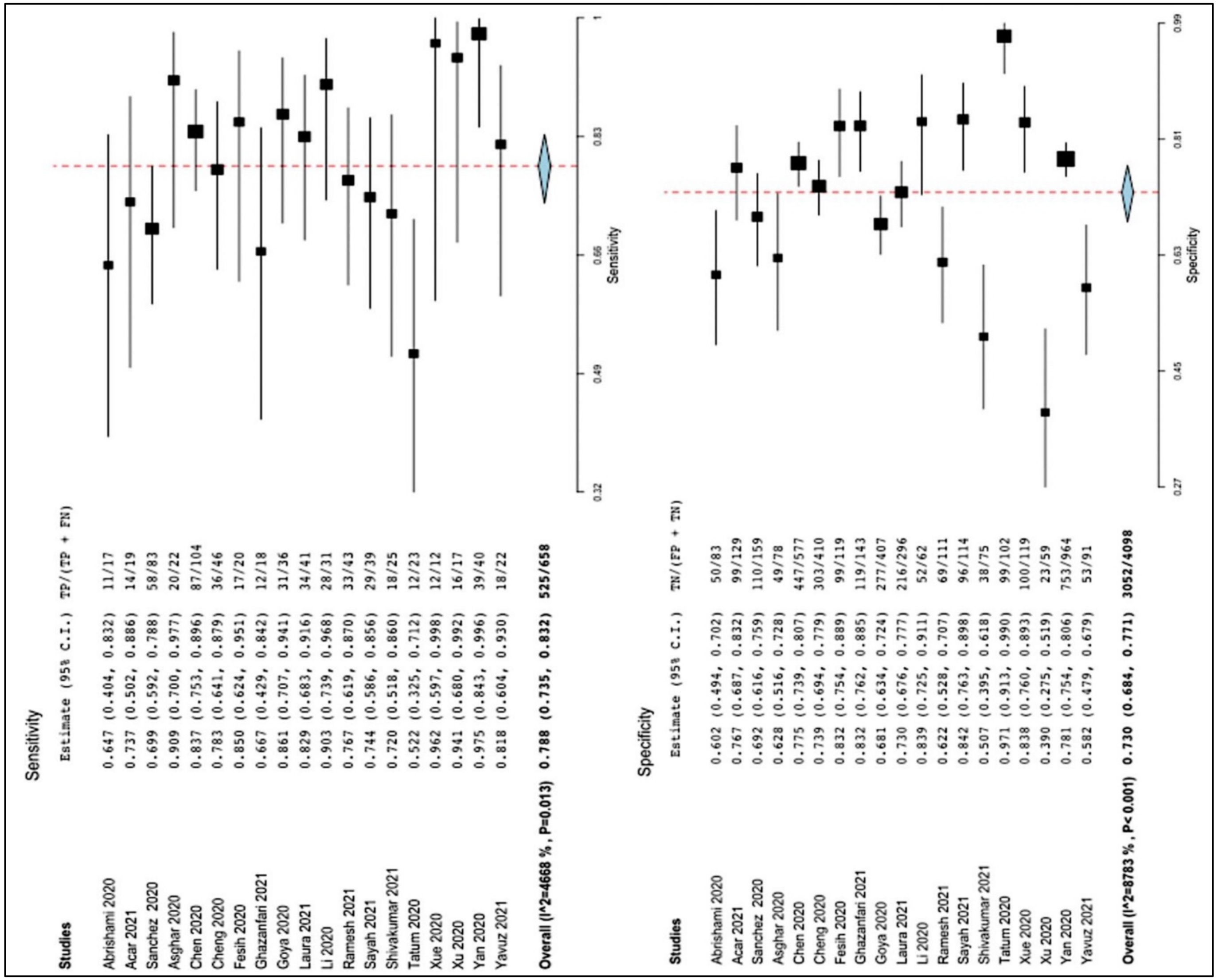

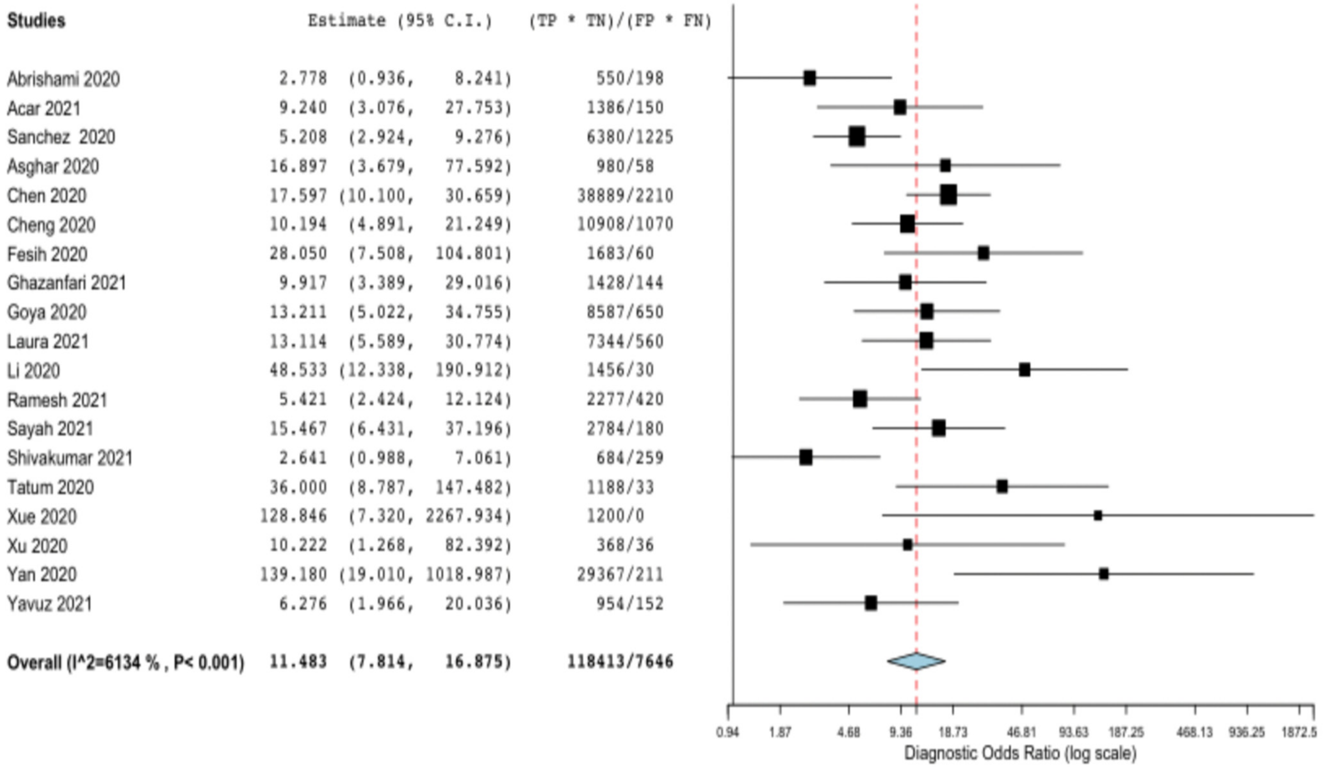

Forest plot of odds of NLR for predicting disease mortality in patients with COVID-19. The pooled OR was 11.483 (95% CI: 7.814 – 16.875).

Figure S4 A: Forest plot showing sensitivity of NLR for predicting disease severity in patients with COVID-19

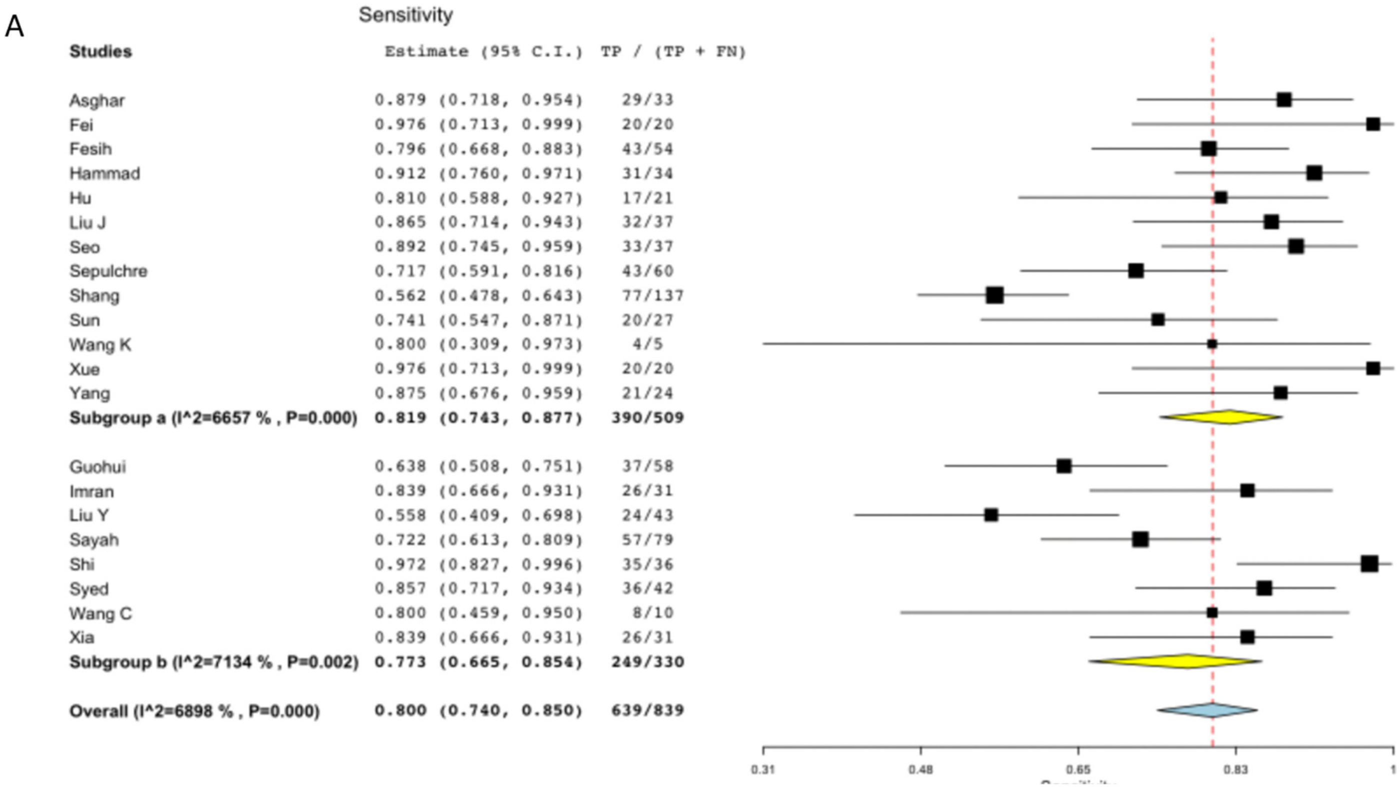

S4A: Forest plot showing sensitivity of NLR for predicting severity in patients with COVID-19

Subgroup a Cut-off <4.5

Subgroup b Cutoff >4.5

Cut-off calculation from the median value of NLR cut-off from our review

Figure S4 B: Forest plot showing specificity of NLR for predicting disease severity in patients with COVID-19

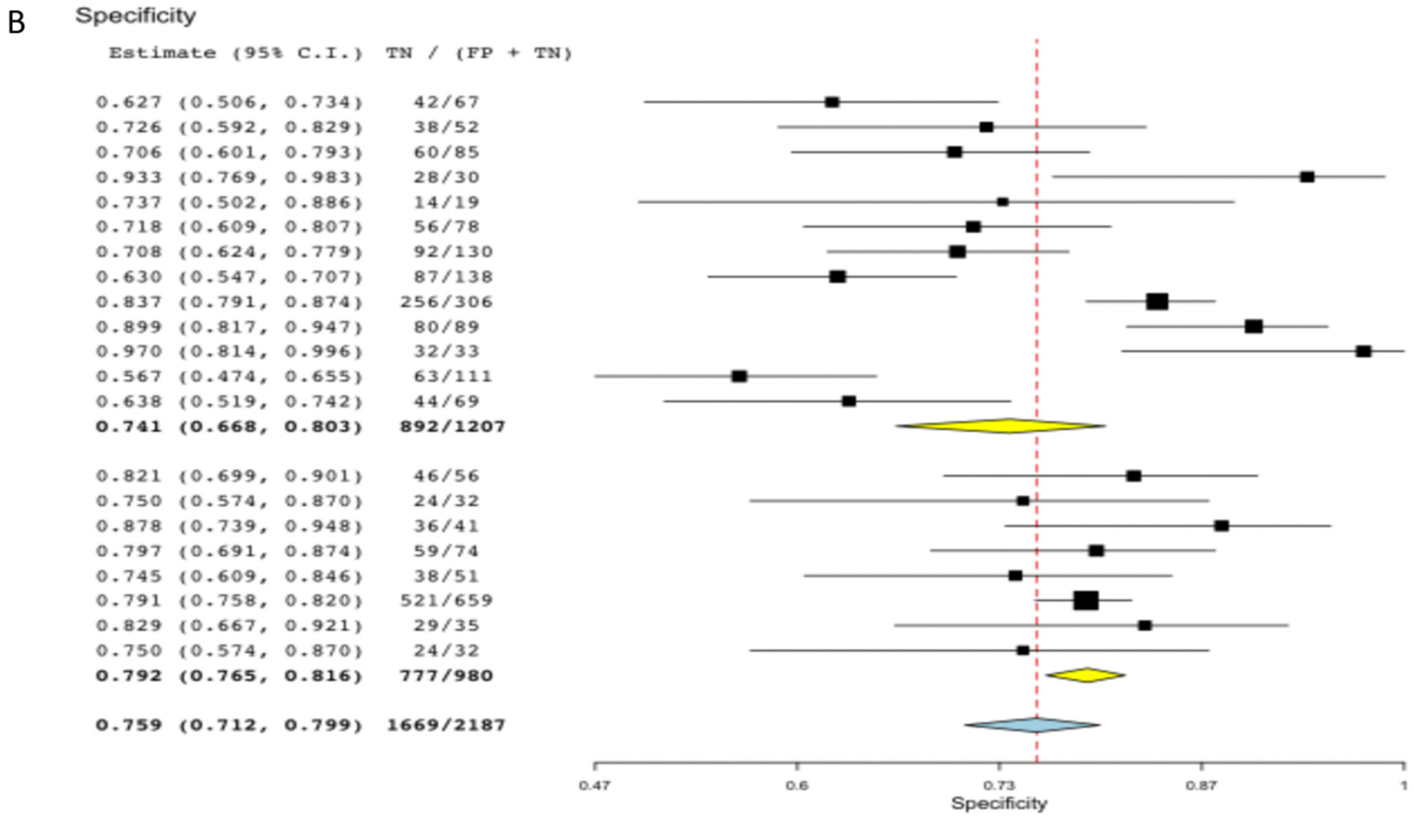

S4B: Forest plot showing specificity of NLR for predicting severity in patients with COVID-19

Subgroup a Cut-off <4.5

Subgroup b Cutoff >4.5

Cut-off calculation from the median value of NLR cut-off from our review

Figure S5: Forest plot showing diagnostic odds ratio (DOR) of NLR for predicting disease severity in patients with COVID-19.

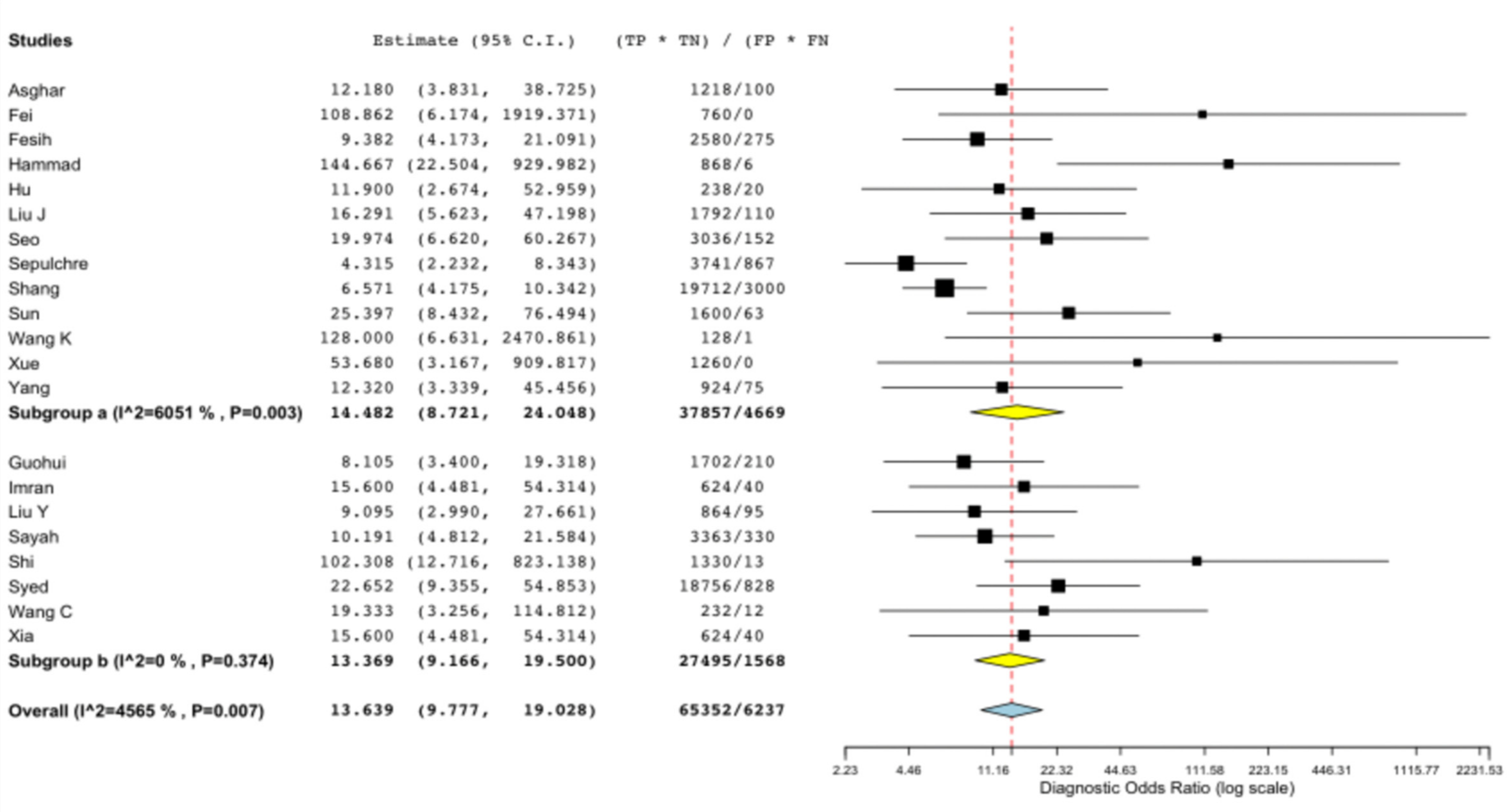

S5: Forest plot showing the diagnostic odds ratio (DOR) of NLR for predicting severity in patients with COVID-19

Subgroup a Cut-off <4.5

Subgroup b Cutoff >4.5

Cut-off calculation from the median value of NLR cut-off from our review

Figure S6: Forest plot showing sensitivity and specificity of NLR for predicting disease Mortality in patients with COVID-19

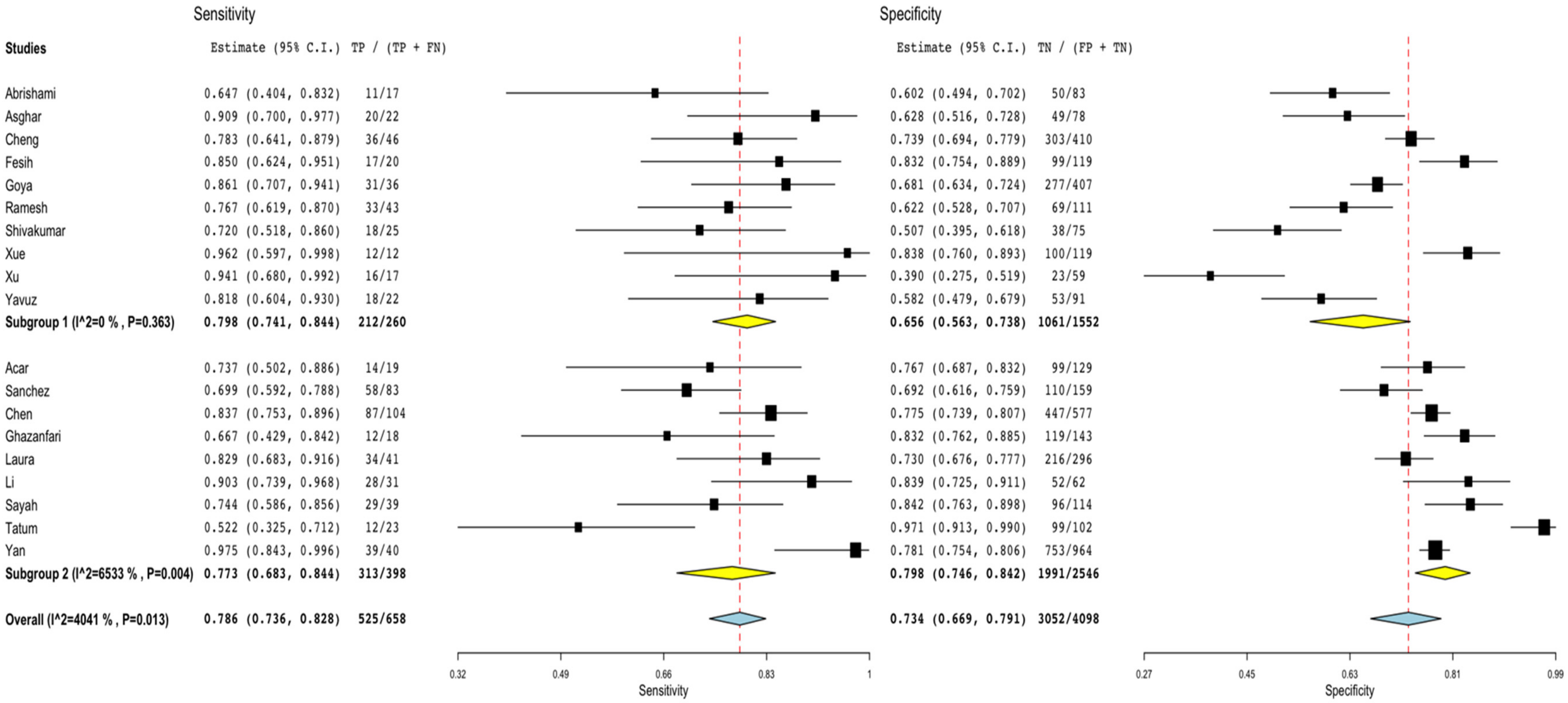

S6: Forest plot showing sensitivity and specificity of NLR for predicting mortality in patients with COVID-19

Subgroup a Cut-off <6.5

Subgroup b Cutoff >6.5

Cut-off calculation from the median value of NLR cut-off from our review

Figure S7: Forest plot showing sensitivity and specificity of NLR for predicting disease Mortality in patients with COVID-19.

Studies	Estimate (95% C.I.)	(TP * TN) / (FP * FN
Abrishami	2.778 (0.936, 8.241)	550/198
Asghar	16.897 (3.679, 77.592)	980/58
Cheng	10.194 (4.891, 21.249)	10908/1070
Fesih	28.050 (7.508, 104.801)	1683/60
Goya	13.211 (5.022, 34.755)	8587/650
Ramesh	5.421 (2.424, 12.124)	2277/420
Shivakumar	2.641 (0.988, 7.061)	684/259
Xue	128.846 (7.320, 2267.934)	1200/0
Xu	10.222 (1.268, 82.392)	368/36
Yavuz	6.276 (1.966, 20.036)	954/152
**Subgroup 1 (I^2=5203 % , P=0.025)**	**8.250 (4.853, 14.025)**	**28191/2903**
Acar	9.240 (3.076, 27.753)	1386/150
Sanchez	5.208 (2.924, 9.276)	6380/1225
Chen	17.597 (10.100, 30.659)	38889/2210
Ghazanfari	9.917 (3.389, 29.016)	1428/144
Laura	13.114 (5.589, 30.774)	7344/560
Li	48.533 (12.338, 190.912)	1456/30
Sayah	15.467 (6.431, 37.196)	2784/180
Tatum	36.000 (8.787, 147.482)	1188/33
Yan	139.180 (19.010, 1018.987)	29367/211
**Subgroup 2 (I^2=6272 % , P=0.004)**	**15.468 (9.195, 26.022)**	**90222/4743**
**Overall (I^2=6230 % , P=0.000)**	**11.515 (7.799, 17.002)**	**118413/7646**

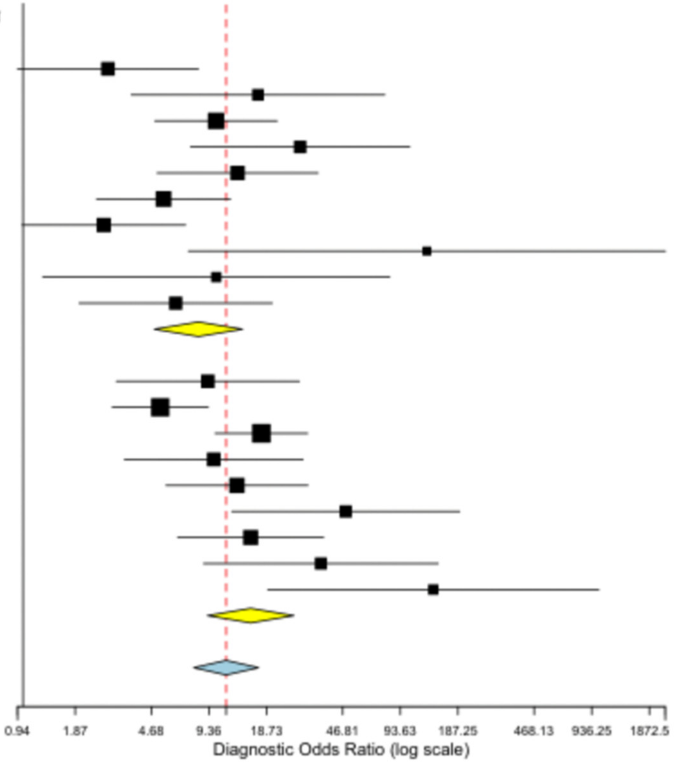

S6: Forest plot showing sensitivity and specificity of NLR for predicting mortality in patients with COVID-19

Subgroup a Cut-off <4.5

Subgroup b Cut-off >4.5

Cut-off calculation from the median value of NLR cut-off from our review

Fig S8: Forest Plot using the random-effects model showing the association between NLR value on admission and all-cause mortality risk.

	Elevated		Normal			Risk Ratio
Study or Subgroup	Events	Total	Events	Total	Weight	M-H, Random, 95% CI
Liu Y et al	26	82	7	163	34.5%	7.38 [3.35, 16.29]
Tatum D et al	14	57	9	62	35.2%	1.69 [0.79, 3.60]
Ullah W et al	4	26	14	141	30.3%	1.55 [0.55, 4.34]
**Total (95% CI)**		**165**		**366**	**100.0%**	**2.74 [0.97, 7.72]**
Total events	44		30			

Heterogeneity: $Tau^2 = 0.65$; $Chi^2 = 8.89$, df = 2 (P = 0.01); $I^2 = 77\%$

Test for overall effect: Z = 1.91 (P = 0.06)

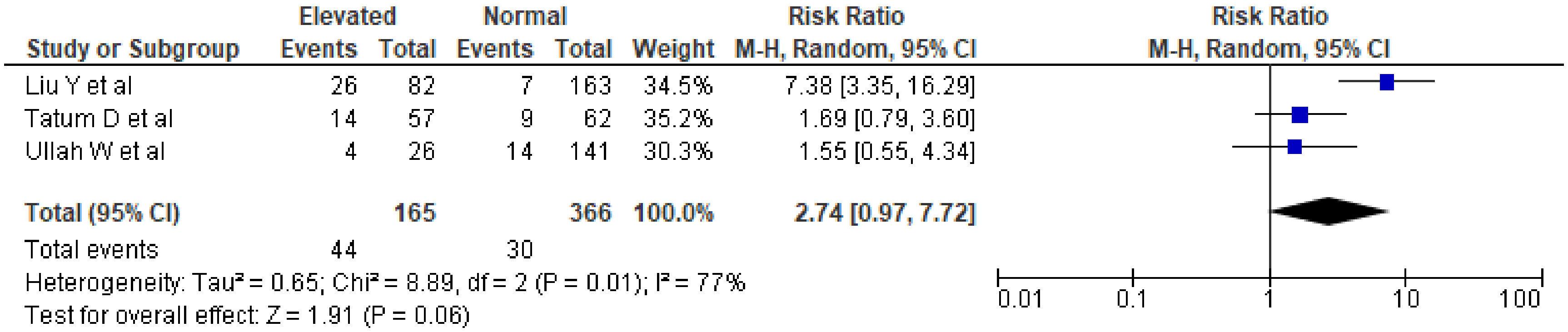

Figure S9: Bubble plot for meta-regression in studies with severity outcome.

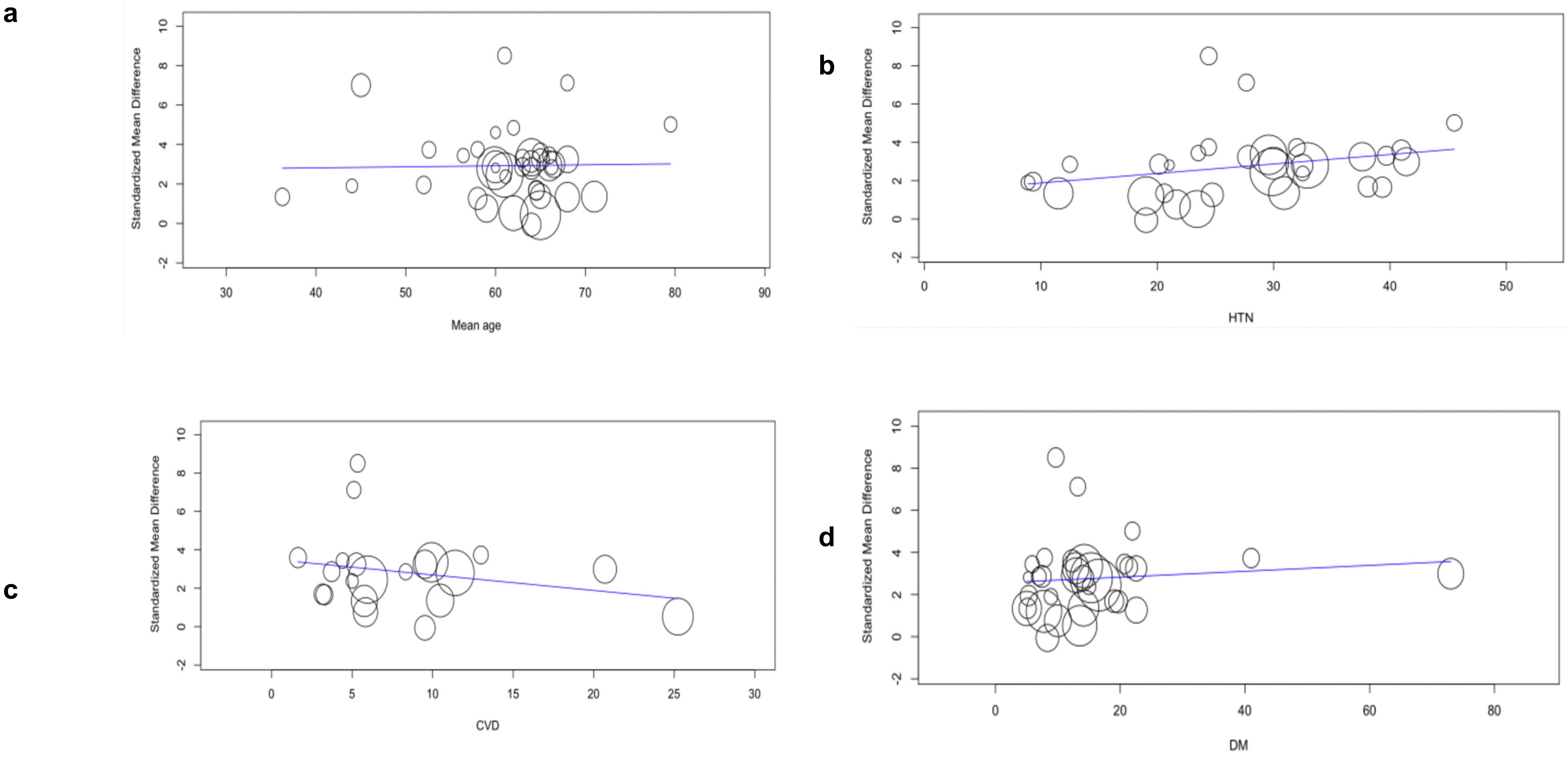

Bubble plot for meta-regression in studies with severity outcome. The association between NLR values on admission and COVID-19 severity was not affected by age (A), Hypertension(B), CVD (C), and Diabetes Meletus(D).

Figure S10: Bubble plot for meta-regression in studies with mortality outcome

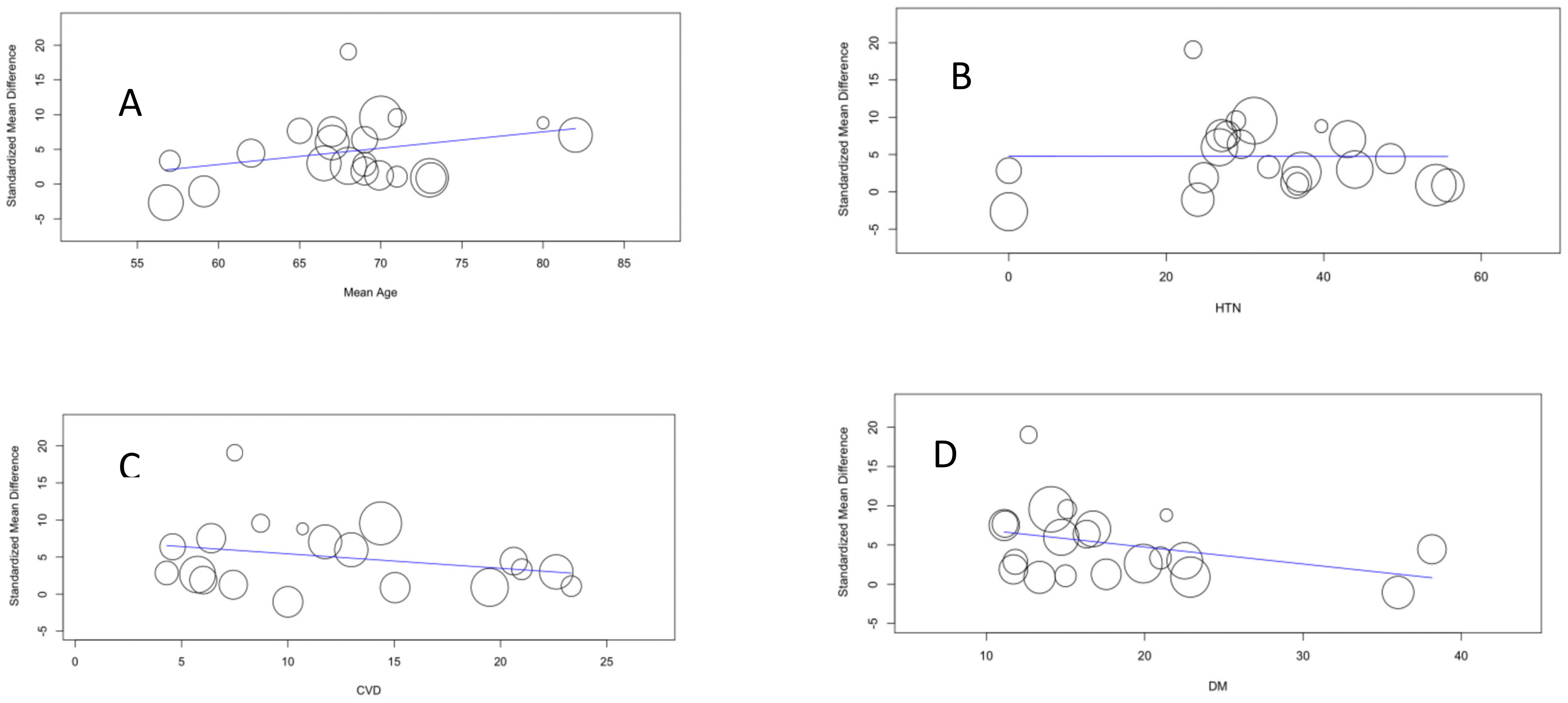

Bubble plot for meta-regression in studies with mortality outcome. The association between NLR values on admission and COVID-19 mortality was not affected by age (A), Hypertension(B), CVD (C), and Diabetes Meletus(D).

Figure S11: Forest plot of studies comparing severe disease with non-severe disease stratified by study design

Study or Subgroup	Severe Mean	Severe SD	Severe Total	Non-Severe Mean	Non-Severe SD	Non-Severe Total	Weight	Std. Mean Difference IV, Random, 95% CI
**2.2.1 Retrospective**								
Asghar et al	9.11	2.26	33	3	1.2	67	2.5%	3.73 [3.06, 4.41]
Bastug A et al	9	8.12	46	2.4	3.17	145	2.6%	1.36 [1.00, 1.72]
Cheng B et al	3.37	0.6	251	2	0.3	205	2.6%	2.80 [2.54, 3.06]
Chen R et al	16.06	3.77	48	3.37	0.76	345	2.5%	8.51 [7.84, 9.18]
Ding X et al	4.8	2.1	15	1.9	0.4	57	2.4%	2.85 [2.11, 3.60]
Fei M et al	6.14	4.75	20	2.66	1.93	52	2.5%	1.16 [0.61, 1.71]
Fu J et al (81)	6.29	3.72	16	2.33	1.22	59	2.5%	1.95 [1.31, 2.59]
Gong J et al	3.7	1.37	28	1.9	0.25	161	2.5%	3.15 [2.63, 3.66]
Hu H et al	10.58	4.16	21	3.14	0.8	19	2.4%	2.38 [1.55, 3.21]
Kazancioglu S et al	4.04	3.5	35	2.31	1.52	85	2.6%	0.76 [0.35, 1.16]
Kong M et al	6.6	1.5	87	3.3	0.4	123	2.6%	3.25 [2.83, 3.67]
Liao D et al	16.02	3.05	86	2.67	0.39	149	2.5%	7.12 [6.42, 7.82]
Lin S et al	9	2.2	46	2.55	0.65	22	2.4%	3.44 [2.66, 4.22]
Liu F et al	3.85	0.75	19	2.7	0.31	115	2.5%	2.86 [2.26, 3.46]
Liu YP et al	16.52	5.17	61	4.3	2.66	23	2.5%	2.61 [1.99, 3.24]
Qin C et al	5.5	1.11	286	3.2	0.51	166	2.6%	2.45 [2.20, 2.70]
Sayed A et al	5.5	1.44	41	2.85	0.16	660	2.5%	7.01 [6.52, 7.49]
Sepulchre E et al	7.25	0.24	60	4	0.62	138	2.5%	6.05 [5.38, 6.73]
Shang W et al	4.76	1.15	139	2.38	0.35	304	2.6%	3.37 [3.07, 3.67]
Wang C et al	29.9	18.7	10	7.93	8.36	35	2.4%	1.90 [1.09, 2.72]
Wang F et al	2.72	0.62	70	1.72	0.22	253	2.6%	2.87 [2.53, 3.22]
Wang K et al	21.39	20.08	5	2.14	0.1	33	2.2%	2.82 [1.66, 3.97]
Wang W et al	9.3	2.32	50	1.83	0.12	73	2.4%	5.02 [4.29, 5.75]
Wang X et al	7.12	1.91	20	2.21	1.39	111	2.5%	3.30 [2.68, 3.93]
Wu S et al	5.8	2.42	67	2.2	0.31	203	2.6%	2.92 [2.55, 3.29]
Xia X et al	12.1	4.83	31	3.27	1.12	32	2.5%	2.51 [1.84, 3.18]
Xie G et al	3.52	1.44	12	2.74	0.32	85	2.5%	1.35 [0.71, 1.98]
Xie L et al	7.9	10.2	51	2.94	1.8	322	2.6%	1.21 [0.90, 1.51]
Xue G et al	6.57	1.66	58	3.03	0.63	56	2.5%	2.78 [2.26, 3.30]
Yang AP et al	20.7	24.1	24	4.8	3.5	69	2.5%	1.26 [0.76, 1.76]
Zhang Y et al	7.58	7.04	31	2.28	1.25	84	2.5%	1.39 [0.94, 1.84]
Zhou Y et al	27.66	11.66	164	3.1	2.41	140	2.6%	2.81 [2.49, 3.13]
Zhu Z et al	5.58	1.96	16	2.75	0.35	111	2.5%	3.73 [3.03, 4.43]
**Subtotal (95% CI)**			**1947**			**4502**	**82.6%**	**3.10 [2.59, 3.62]**
Heterogeneity: $Tau^2 = 2.20$; $Chi^2 = 1289.43$, df = 32 (P < 0.00001); $I^2 = 98\%$								
Test for overall effect: Z = 11.76 (P < 0.00001)								
**2.2.2 Prospective**								
Guner R et al	9.12	5.6	50	2.5	4.6	172	2.6%	1.36 [1.02, 1.70]
Hammad R et al	10	2.57	34	1.23	0.2	30	2.3%	4.61 [3.65, 5.57]
Liu J et al	8.83	1.88	79	3.11	0.76	43	2.5%	3.60 [3.01, 4.19]
Ok F et al	6.1	3.78	54	2.46	1.7	85	2.6%	1.34 [0.96, 1.72]
Sayah W et al	8.2	2.05	60	3.5	0.58	73	2.5%	3.24 [2.72, 3.76]
Shi S et al	6.78	2.01	36	2.2	0.32	51	2.5%	3.46 [2.78, 4.13]
Sun S et al	8.71	2.65	27	2.41	0.28	89	2.4%	4.85 [4.09, 5.62]
**Subtotal (95% CI)**			**340**			**543**	**17.4%**	**3.17 [2.13, 4.21]**
Heterogeneity: $Tau^2 = 1.87$; $Chi^2 = 160.77$, df = 6 (P < 0.00001); $I^2 = 96\%$								
Test for overall effect: Z = 5.97 (P < 0.00001)								
**Total (95% CI)**			**2287**			**5045**	**100.0%**	**3.11 [2.65, 3.57]**
Heterogeneity: $Tau^2 = 2.11$; $Chi^2 = 1465.31$, df = 39 (P < 0.00001); $I^2 = 97\%$								
Test for overall effect: Z = 13.27 (P < 0.00001)								
Test for subgroup differences: $Chi^2 = 0.01$, df = 1 (P = 0.91), $I^2 = 0\%$								

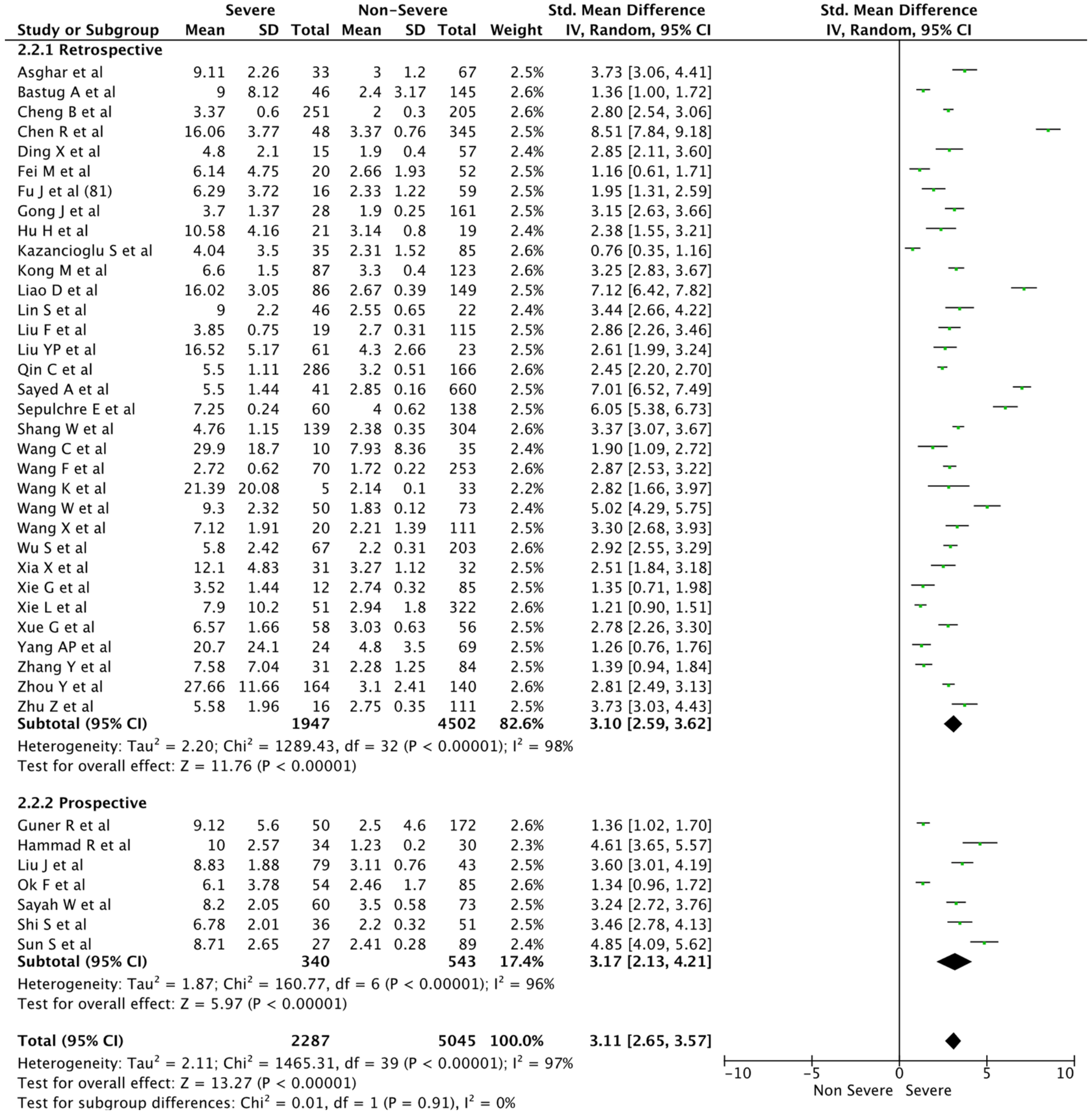

Figure S12 : Forest plot of studies comparing non-survivors with survivors, stratified by study design

Study or Subgroup	Non-survivors Mean	Non-survivors SD	Non-survivors Total	Survivors Mean	Survivors SD	Survivors Total	Weight	Std. Mean Difference IV, Random, 95% CI
**2.1.1 Retrospective**								
Wang X et al	15.01	5.01	12	1.95	0.19	119	4.5%	8.81 [7.57, 10.04]
Yan X et al	49.06	10.99	40	4.11	0.94	964	4.5%	19.07 [18.17, 19.96]
Luo X et al	8.17	0.79	84	2.96	0.41	214	4.5%	9.55 [8.74, 10.36]
Zhang N et al	18.7	16.6	10	8.4	7.5	50	4.5%	1.07 [0.36, 1.78]
Li L et al	4.25	0.95	25	2.3	0.55	68	4.5%	2.85 [2.23, 3.47]
Ye W et al	14.94	4.51	52	2.88	0.82	297	4.5%	6.38 [5.82, 6.93]
Huang J et al	13.3	14.3	16	3.3	4.3	283	4.6%	1.89 [1.36, 2.42]
Asghar et al	12.65	10.37	22	5.45	5.4	78	4.6%	1.05 [0.56, 1.55]
Sepulchre E et al	7	1.75	43	4	0.33	155	4.6%	3.47 [2.99, 3.95]
Yavuz B et al	9.9	10.5	22	4.5	4.6	91	4.6%	0.87 [0.39, 1.35]
BG et al	4.87	3.7	25	8.88	3.84	75	4.6%	−1.05 [−1.52, −0.57]
Chen F et al	12.27	2.79	104	3.38	0.69	577	4.6%	7.05 [6.62, 7.48]
Zhang S et al	10.99	2.21	96	3.91	0.78	420	4.6%	5.98 [5.55, 6.41]
Güneysu F et al	2.88	0.54	85	6.43	1.8	84	4.6%	−2.67 [−3.08, −2.25]
Yang Q et al	6.18	2.3	50	2.98	0.63	176	4.6%	2.63 [2.23, 3.03]
Cheng B et al	13.3	14.9	51	4.1	2.9	67	4.6%	0.91 [0.53, 1.29]
Chen L et al	11	2.33	208	3	0.33	1651	4.6%	9.54 [9.21, 9.88]
**Subtotal (95% CI)**			**945**			**5369**	**77.3%**	**4.54 [2.45, 6.63]**
Heterogeneity: $Tau^2 = 19.23$; $Chi^2 = 4830.98$, df = 16 (P < 0.00001); $I^2 = 100\%$								
Test for overall effect: Z = 4.26 (P < 0.0001)								
**2.1.2 Prospective**								
Abrishami A et al	5.81	1.9	17	3.02	0.34	83	4.5%	3.34 [2.64, 4.05]
Goya R L et al	16.9	5.25	36	4.2	0.93	465	4.5%	7.66 [7.08, 8.25]
Sanchez A et al	17.66	2.71	83	8.31	1.47	111	4.6%	4.45 [3.92, 4.98]
Acar et al	27.15	14.71	19	9.4	13.85	129	4.6%	1.27 [0.76, 1.77]
Chen R et al	13.45	2.37	103	3.71	0.87	445	4.6%	7.54 [7.04, 8.03]
**Subtotal (95% CI)**			**258**			**1233**	**22.7%**	**4.85 [2.33, 7.38]**
Heterogeneity: $Tau^2 = 8.24$; $Chi^2 = 416.20$, df = 4 (P < 0.00001); $I^2 = 99\%$								
Test for overall effect: Z = 3.76 (P = 0.0002)								
**Total (95% CI)**			**1203**			**6602**	**100.0%**	**4.61 [2.89, 6.33]**
Heterogeneity: $Tau^2 = 16.82$; $Chi^2 = 5318.92$, df = 21 (P < 0.00001); $I^2 = 100\%$								
Test for overall effect: Z = 5.26 (P < 0.00001)								
Test for subgroup differences: $Chi^2 = 0.03$, df = 1 (P = 0.85), $I^2 = 0\%$								

Std. Mean Difference
IV, Random, 95% CI

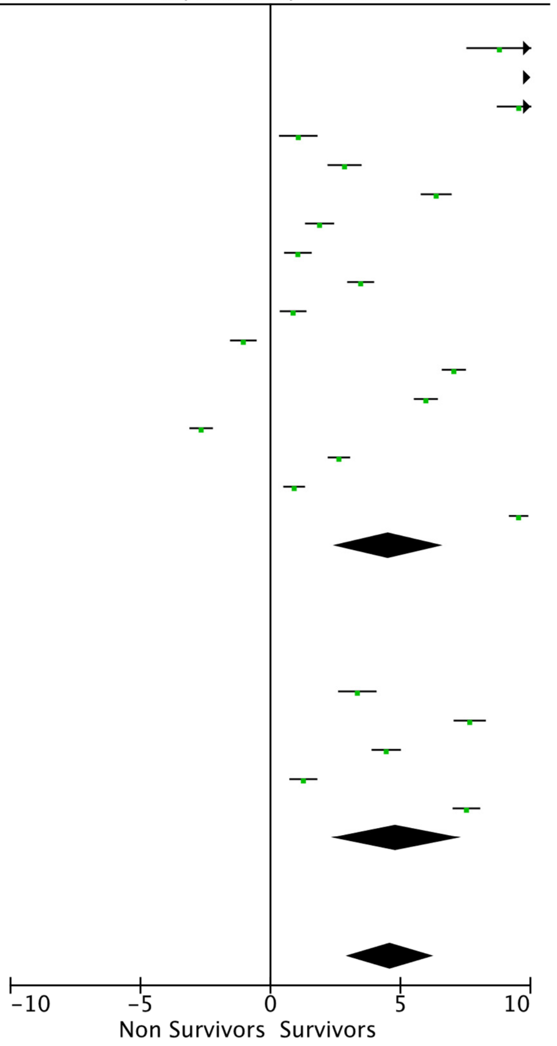

Supplement: Supplementary file 1 [file vaccines-10-01233-s001.zip › vaccines-1811881-supplementary.pdf]
